# Integrated multi-omics unveil the impact of *H*-phosphinic analogs of glutamate and α-ketoglutarate on *Escherichia coli* metabolism

**DOI:** 10.1016/j.jbc.2024.107803

**Published:** 2024-09-21

**Authors:** Fabio Giovannercole, Luís Gafeira Gonçalves, Jean Armengaud, Ana Varela Coelho, Alex Khomutov, Daniela De Biase

**Affiliations:** 1 Department of Medico-Surgical Sciences and Biotechnologies, Sapienza University of Rome, Latina, Italy; 2 Instituto de Tecnologia Química e Biológica, António Xavier, Universidade Nova de Lisboa, Oeiras, Portugal; 3 Département Médicaments et Technologies pour la Santé (DMTS), Université Paris-Saclay, CEA, INRAE, SPI, Bagnols-sur-Ceze, France; 4 Engelhardt Institute of Molecular Biology, Russian Academy of Sciences, Moscow, Russia

**Keywords:** desmethylphosphinothricin, phosphinates, glutamate, multi-target drug, multi-omics, NMR, LC-MS, nitrogen starvation

## Abstract

Desmethylphosphinothricin (*L*-Glu-γ-P_H_) is the *H*-phosphinic analog of glutamate with carbon-phosphorus-hydrogen (C-P-H) bonds. In *L*-Glu-γ-P_H_ the phosphinic group acts as a bioisostere of the glutamate γ-carboxyl group allowing the molecule to be a substrate of *Escherichia coli* glutamate decarboxylase, a pyridoxal 5′-phosphate-dependent α-decarboxylase. In addition, the *L*-Glu-γ-P_H_ decarboxylation product, GABA-P_H_, is further metabolized by bacterial GABA-transaminase, another pyridoxal 5′-phosphate-dependent enzyme, and succinic semialdehyde dehydrogenase, a NADP^+^-dependent enzyme. The product of these consecutive reactions, the so-called GABA shunt, is succinate-P_H_, the *H*-phosphinic analog of succinate, a tricarboxylic acid cycle intermediate. Notably, *L*-Glu-γ-P_H_ displays antibacterial activity in the same concentration range of well-established antibiotics in *E. coli*. The dipeptide *L*-Leu-Glu-γ-P_H_ was shown to display an even higher efficacy, likely as a consequence of an improved penetration into the bacteria. Herein, to further understand the intracellular effects of *L*-Glu-γ-P_H_, ^1^H NMR-based metabolomics, and LC-MS-based shotgun proteomics were used. This study included also the keto-derivative of *L*-Glu-γ-P_H,_ α-ketoglutarate-γ-P_H_ (α-KG-γ-P_H_), which also exhibits antimicrobial activity. *L*-Glu-γ-P_H_ and α-KG-γ-P_H_ are found to similarly impact bacterial metabolism, although the overall effect of α-KG-γ-P_H_ is more pervasive. Notably, α-KG-γ-P_H_ is converted intracellularly into *L*-Glu-γ-P_H_, but the opposite was not found. In general, both molecules impact the pathways where aspartate, glutamate, and glutamine are used as precursors for the biosynthesis of related metabolites, activate the acid stress response, and deprive cells of nitrogen. This work highlights the multi-target drug potential of *L*-Glu-γ-P_H_ and α-KG-γ-P_H_ and paves the way for their exploitation as antimicrobials.

Phosphonic and phosphinic acids are a group of uncommon natural products with one or more carbon-phosphorus (C-P) bond(s), resistant to hydrolysis by phosphatases and phosphodiesterases as well as to chemical treatments, such as boiling and acid or base treatments (1). Phosphonates contain one C-P bond, whereas phosphinates can present either two C-P bonds or one C-P and one P-H bond occurring on the same phosphorous atom (1, 2). Due to the C-P bond(s), phosphonates and phosphinates structurally resemble chemical groups such as phosphate esters, carboxyl groups, and tetrahedral intermediates occurring during the transformation of carboxylic acids derivatives. This feature allows phosphonates and phosphinates to be easily recognized by the cellular enzymes involved in the biochemical transformations of these chemical groups (1, 2, 3). When this occurs, the inability to hydrolyze the C-P bond(s) impedes the completion of the reaction, which may even result in irreversible enzymatic inhibition (1, 2). Due to the abovementioned properties and given the ubiquitous involvement of the targeted enzymes in metabolic and signaling pathways, phosphonates and phosphinates have a large application as bioactive molecules for the treatment of several human diseases, including microbial infections (4).

Some naturally produced phosphinates and phosphonates chemically resemble proteinogenic amino acids. A remarkable example is given by *L-*Phosphinothricin [*L*-2-amino-4-(hydroxymethyl-phosphinyl)butanoic acid, also known as glufosinate; *L-*PT in Fig. 1], a phosphinic analog of glutamate with its phosphinic moiety (C-P-CH_3_) replacing the γ-carboxyl group (1). *L-*PT was initially discovered and isolated from cultures of *Streptomyces hygroscopicus* and *Streptomyces viridochromogenes* (2, 5, 6). *L-*PT irreversibly inhibits the enzyme glutamine synthetase (GS), which catalyzes the ATP-dependent formation of glutamine from glutamate and ammonia. When in the active site of GS, *L-*PT becomes phosphorylated on the γ-phosphinic moiety, thus leading to a species that mimics the γ-glutamyl phosphate intermediate normally occurring during the catalysis. The reaction however cannot proceed further, and the enzyme-bound intermediate remains in the active site where it is protected from hydrolysis (7, 8). Since GS plays a key role in nitrogen assimilation, its inhibition causes the accumulation of toxic levels of ammonia which inevitably leads to cell death (9, 10). For this reason, *L*-PT has bactericidal, fungicidal, and herbicidal properties and nowadays is largely employed in the form of the broad-spectrum herbicides Bialaphos and Phosalacine, in which *L-*PT is enzymatically condensed with either two *L*-alanine residues or *L*-alanine and *L*-leucine residues, respectively (1) (Fig. 1). The effect of the herbicidal activity of *L*-PT has been investigated in further detail on buffalo grasses using an untargeted metabolomics approach which confirmed that *L*-PT upregulates free amino acids pool, sugar-phosphate and amino acid metabolism (10). A recent report also showed the efficacy of Bialaphos and of a dipeptide *L*-Leucyl-*L*-PT on clinical isolates of the human pathogen *Klebsiella pneumoniae*, resistant to more than 20 commercial antibiotics belonging to different classes (11).

**Figure 1:**
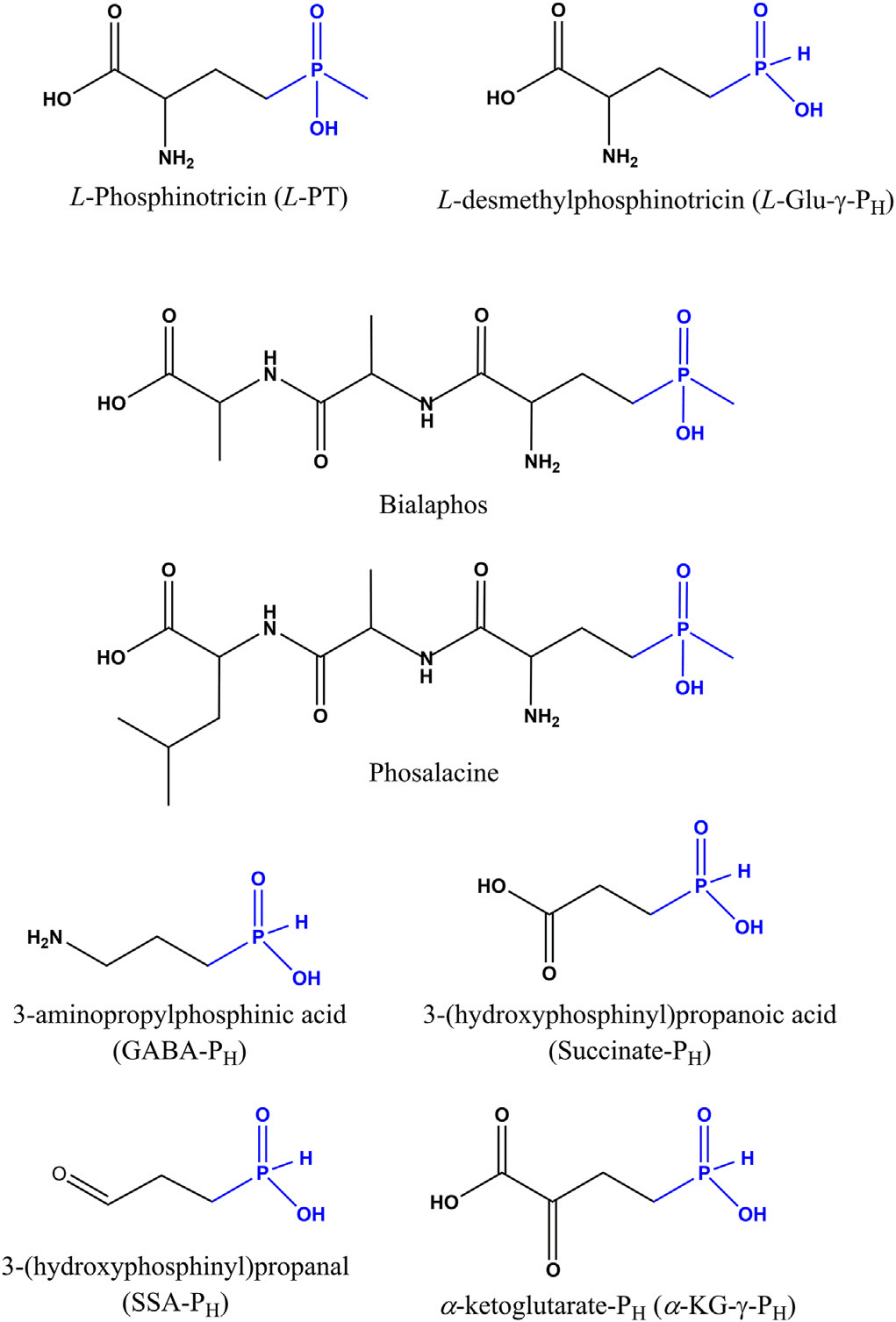
**Structural formula of phosphinates.***L*-phosphinotricin (*L*-PT) and the natural tripeptides that derive from it (Bialaphos and Phosalacine) are shown along with the *H*-phosphinic compound *L*-Glu-γ-P_H_ (*L*-desmethylphosphinotricin) and its derivatives obtained either by enzymatic decarboxylation (GABA-P_H_), followed by transamination (SSA-P_H_) and oxidation (succinate-P_H_), or by either transamination or oxidative deamination (α-KG-γ-P_H_).

The work of independent groups brought to the identification of the *L-*PT and Bialaphos biosynthetic gene cluster in *S. hygroscopicus* and *S. viridochromogenes* (2, 12, 13, 14, 15, 16, 17, 18, 19). In the attempt to dissect the chemical steps occurring during the complex biosynthesis of *L-*PT*,* the intermediate desmethylphosphinothricin [*L*-2-amino-4-(hydroxy)phosphinylbutyric acid; initially named MP101 or DMPT; hereafter and in Fig. 1 *L-*Glu-γ-P_H_] was discovered and isolated. *L-*Glu-γ-P_H_ lacks the methyl group present in *L*-PT (C-P-CH_3_) which is instead replaced by a hydrogen (C-P-H) (20, 21). *L-*Glu-γ-P_H_ was found to inhibit the growth of the producing strains at 10 μg/ml (*i.e.* 60 μM), as well as to display negligible toxic effects when administered in rats and mice (20, 22). In addition to *S. hygroscopicus* and *S. viridochromogenes*, *L-*Glu-γ-P_H_ was also isolated from *Nonomureae* sp. NRLL B-24552 (23).

It was reported that *L*-Glu-γ-P_H_ is decarboxylated by the *Escherichia coli* enzyme glutamate decarboxylase (*Ec*GadB; E.C. 4.1.1.15), a pyridoxal 5′-phosphate (PLP)-dependent enzyme involved in the acid stress response in many enteropathogens (24, 25, 26, 27). It was shown that the chemical contacts that stabilize the PLP-*L*-Glu adduct are conserved in the PLP-*L*-Glu-γ-P_H_ adduct, and this allowed to conclude that, due to its single charge flatted tetrahedral structure, the *H*-phosphinic moiety of *L*-Glu-γ-P_H_ is a bioisostere of *L*-Glu carboxyl group in γ-position (26). Furthermore, the *Ec*GadB-dependent decarboxylation of *L*-Glu-γ-P_H_ yielded the *H*-phosphinic analog of GABA (3-aminopropylphosphinic acid, 3-APPA; GABA-P_H_ in Fig. 1), which could be further transformed into the *H*-phosphinic analog of succinic semialdehyde [3-(hydroxyphosphinyl)propanal, succinic semialdehyde-P_H_; SSA-P_H_ in Fig. 1] and then into succinate-P_H_ (Fig. 1) by GABase, a commercial crude preparation of *Pseudomonas fluorescens* containing GABA-transaminase and succinic semialdehyde dehydrogenase (26). Taken together, the above results suggest that *L-*Glu-γ-P_H_ may be a better substrate than *L-*PT in targeting glutamate-dependent reactions intracellularly. This was further put forward in a recently published study where a Glu-γ-P_H_ dipeptide derivative was shown to effectively inhibit the growth of both Gram-negative and Gram-positive model microorganisms (28). The inhibition of the growth of *E. coli* K12 MG1655 strain, with a minimum inhibitory concentration (MIC) comparable to that of well-known antibiotics, such as ampicillin (42 μM vs 11 μM, respectively) under the same experimental conditions (28), makes Glu-γ-P_H_ a promising novel antibacterial, given that it is currently recognized that inhibiting multiple targets may be a useful feature to minimize the development of antibiotic resistance in bacteria (29) and given that *L-*Glu-γ-P_H_ shows limited effect on eukaryotic cells (22). In fact, thanks to the two pharmacophores, the α-carbon moiety and the *H*-phosphinic group, *L-*Glu-γ-P_H_ may undergo transformation to phosphorus-containing metabolites, or it may act as potent enzymatic inhibitor in ATP-dependent transformations where the distal carboxyl group of glutamate is involved (28). The former would result in the production of phosphinic derivatives that could affect central microbial metabolism, while the latter would lead to enzymatic inhibition as observed in GS with *L-*PT (7). It is important to recall that the geometry of the H_3_C-phosphinic group of *L*-PT is different from that of the flattened tetrahedral *H*-phosphinic moiety because of the size of the methyl group and therefore H_3_C-phosphinic group cannot be considered as a bioisostere of the carboxyl group. As a matter of fact, *L*-PT was shown not to be a competitive inhibitor of *E. coli* glutamate decarboxylase (30) and to display a K_i_ 0.6 μM against *E. coli* GS, whereas the phosphonic analog of glutamate having two hydroxyl groups displays a K_i_ of 50 μM (28).

To date, very little is known about the biological effect of *L-*Glu-γ-P_H_ on *E. coli*. As a result, this work wants to cover this lack of knowledge by using a modern untargeted multi-omics approach, rarely used to study the effect of this class of molecules at the cellular level, in particular employing ^1^H NMR-based metabolomics combined with liquid chromatography-tandem mass spectrometry (LC-MS/MS) based proteomics. The study also included the *H-*phosphinic analog of α-ketoglutarate, (2-oxo-4-*H*-phosphinyl)butyric acid; α-KG-P_H_ in Fig. 1), *i.e.*, the natural precursor of *L-*Glu-γ-P_H_ in *L*-PT biosynthesis (2), which is herein shown to display an inhibitory effect on bacterial growth similar to that of *L*-Glu-γ-P_H_. A patent IT 102016000098005 has been granted, which includes α-KG-γ-PH, one of the compounds investigated in detail in this study (https://www.uniroma1.it/en/brevetto/102016000098005). Furthermore, one of the outcomes generated by the multi-omics analysis was experimentally corroborated through phenotypic assays.

## Results

### α-KG-γ-P_H_ displays bacteriostatic activity on *E. coli*

*L*-Glu-γ-P_H_ was previously shown to display a bacteriostatic effect in *E. coli* being able to inhibit cell growth with an MIC_90_ (*i.e.* the minimum concentration able to inhibit 90% of the cell growth) of 7 μg/ml, *i.e.*, 42 μM (Fig. S1*A*) (26). In this work, the effects on *E. coli* metabolism of *L*-Glu-γ-P_H_ and its keto-acid derivate, *i.e.* α-ketoglutarate-γ-P_H_ (α-KG-γ-P_H_), which arises from the oxidative deamination or transamination of *L*-Glu-γ-P_H_ (31, 32), were investigated. In fact, α-KG-γ-P_H_ also exhibited a bacteriostatic effect with an MIC_90_ of 13 μg/ml, *i.e.*, 78 μM (Fig. S1*B*). It was therefore decided to include in the study also α-KG-γ-P_H_, owing to the possible direct interconversion between the two molecules and because this could help to better understand their cellular transformations.

### The effects of *L*-Glu-γ-P_H_ and α-KG-γ-P_H_ on the *E. coli* metabolome

Bacteria were grown in chemically defined minimal medium EG, pH 7.0, to an optical density at 600 nm (OD_600_) of 1.0 in the presence of either *L*-Glu-γ-P_H_ or α-KG-γ-P_H_, at a sub-inhibitory concentration (MIC_50_), which was chosen because it still allowed growth, though at lower rate, as reported in Experimental procedures (section “Sample collection for metabolomic and proteomic analysis”). The control condition consisted of growing bacteria in the absence of either compound (untreated). Pellets from 10 biological replicates for each condition (*i.e.* untreated group, *L*-Glu-γ-P_H_ - and α-KG-γ-P_H_ -treated groups) were collected and processed for metabolomics and proteomics analyses, as schematically depicted in Fig. S2. Based on the analysis of the entire ^1^H NMR spectra, one sample from the *L*-Glu-γ-P_H_ -treated group projected beyond the 95% confidence interval (CI) and was therefore excluded from all downstream analyses.

**Figure 2:**
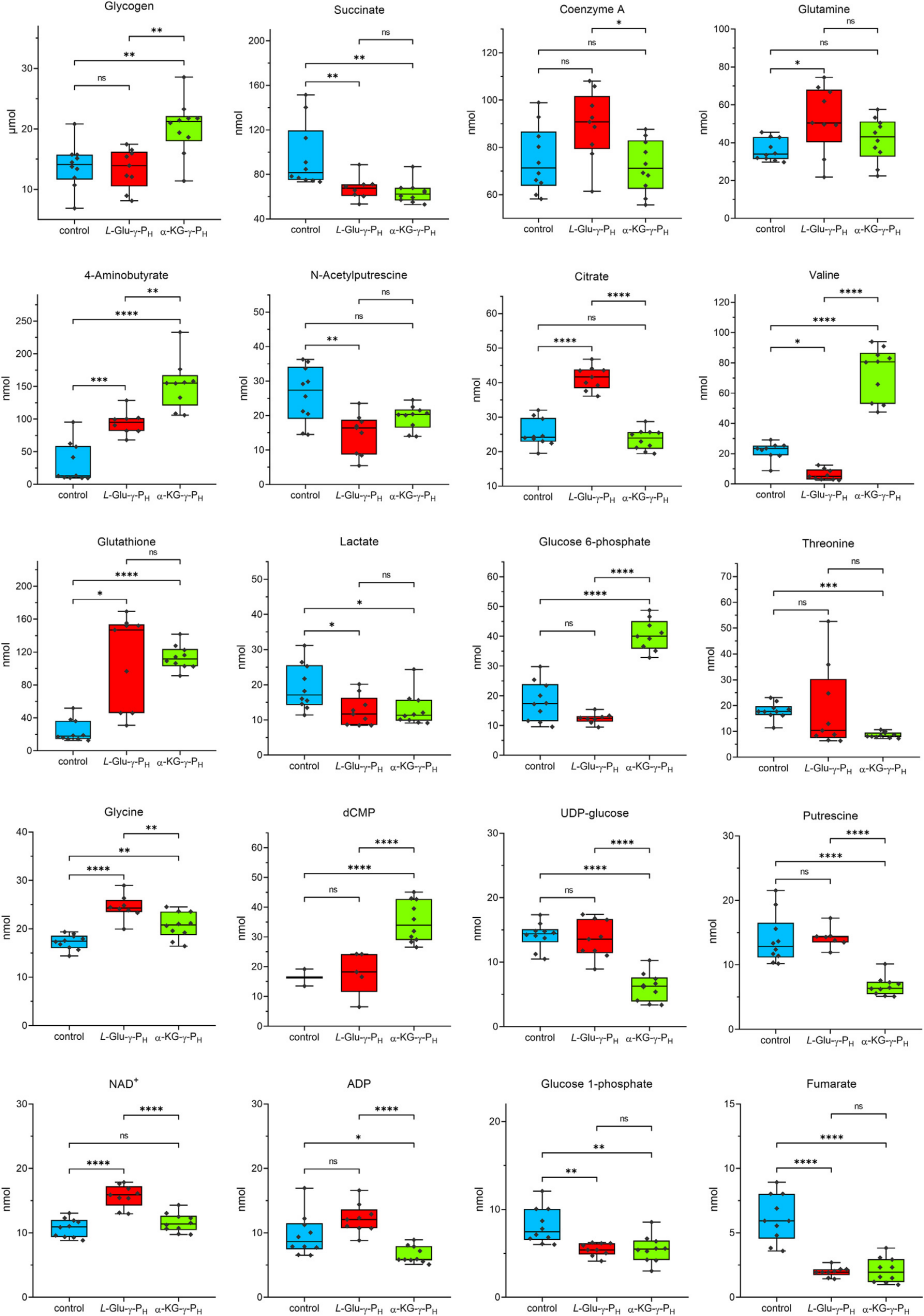
**Univariate analysis of the 20 most abundant and significantly affected metabolites.** The metabolites include those most abundant in the control group (*cyan*) whose concentration is significantly altered in the *L*-Glu-γ-P_H_-(*red*) and/or α-KG-γ-P_H_ (*green*) treated cells. Data are plotted as box-and-whisker plot. Values above/below 3.5 times the interquantile range were considered outliers and excluded. Statistical analysis was performed with one-way ANOVA or Kruskal-Wallis’s test, and *p*-value was adjusted with the *post hoc* Tukey’s HSD test or Mann-Whitney U’s test (with the Bonferroni’s correction). *p* > 0.05 (ns); *p* ≤ 0.05 (∗); *p* ≤ 0.01 (∗∗); *p* ≤ 0.001 (∗∗∗); *p* ≤ 0.0001 (∗∗∗∗).

In the ^1^H NMR spectra of the control group, 59 metabolites were identified and quantified. The same metabolites, with the inclusion of aspartate, cytidine, and uracil, for a total of 62 metabolites, were also detected in the *L*-Glu-γ-P_H_ - and α-KG-γ-P_H_ -treated groups (Table S1). Approximately 10% of the NMR peaks remained unassigned because no corresponding metabolites were present in the databases. In all samples, glycogen was found to be the most abundant, *i.e.* in the μmoles range. Fig. 2 shows the boxplots of the top 20 metabolites that, amongst the most abundant in the untreated group, display statistically significant differences in one or both treated groups.

**Figure 3:**
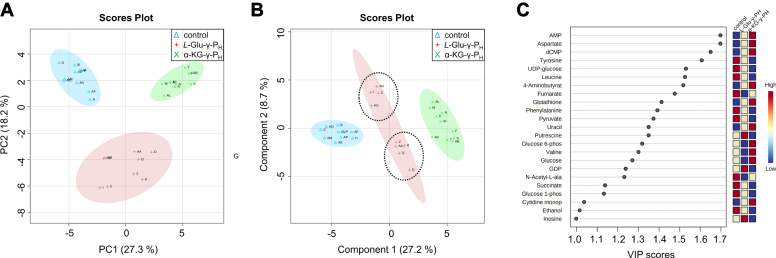
**PCA, PLS-DA and VIP scores of the intracellular metabolites identified by**^**1**^**H NMR.***A*, PCA and *B*, PLS-DA scores plot depicting the projection of control (*n* = 10, cyan triangle), and of *L-*Glu-γ-P_H_ (*n* = 9, *red cross*) and α-KG-γ-P_H_ (*n* = 10, *green* X) treated samples along PC1 and PC2 (*A*), or (*B*). Shaded ellipse areas indicate the 95% confidence interval based on the data points for individual groups. The letters in the score plots represent the biological replicates. Control: A, B, G, H, AE, AF, AM, AN, AO, AP; *L*-Glu-γ-P_H_: C, D, I, Q, R, Z, AA, AG, AH; α-KG-γ-P_H_: E, F, M, N, S, T, AB, AC, AI, AL. In PLS-DA, the two dashed-ellipse areas of the *L-*Glu-γ-P_H_ treated samples define the two sub-clusters. Q2 of first component is 0.92057, while Q2 of the second component is 0.93525. The permutation test value (*p*) is 0.0015. *C*, most influent metabolites ranked according to the VIP scores of the first component of the PLS-DA model. Only metabolites with a VIP score > 1 are reported.

To globally assess the effect of the exposure and adaptation of the *E. coli* metabolome to the *H*-phosphinic compounds, the detected metabolites were subjected to a Principal Component Analysis (PCA) (Fig. 3*A*). Glycogen, which significantly increased in the α-KG-γ-P_H_ -treated group (Table S1), was omitted because it negatively affected the data scaling. The first and the second components (*i.e.* PC1 and PC2) accounted for 27.3% and 18.2% of the total variance, respectively. This unsupervised analysis provided a clear separation of the three experimental groups. This first evidence indicated that *L*-Glu-γ-P_H_ and α-KG-γ-P_H_ have a different effect on the *E. coli* metabolome. Unlike PC2, the projection along PC3 did not provide a significant separation of the three groups as it only described the variance (12.1%) among samples of the same cluster (Table S1).

Like PCA, the metabolites discriminated *via* the scores plot of the supervised multivariate statistical analysis Partial Least Square Discriminating Analysis (PLS-DA) showed that the *L*-Glu-γ-P_H_ -treated group samples could be separated into two sub-clusters (Fig. 3*B*). The PLS-DA model allowed capturing the most important metabolites responsible for the projection of the three groups along the first and the second components of the model. Metabolites were ranked according to the Variable Importance Projection (VIP) score, as shown in Fig. 3*C* for the first component (and in Table S1 for all components). Based on the observed trends, the metabolites were clustered as follows: *i)* metabolites increased in both the *L*-Glu-γ-P_H_- and α-KG-γ-P_H_ -treated groups; *ii)* metabolites decreased in both the *L*-Glu-γ-P_H_ - and α-KG-γ-P_H_ -treated groups; *iii)* metabolites with opposite trend between the *L*-Glu-γ-P_H_ - and α-KG-γ-P_H_ -treated groups.

AMP, aspartate, dCMP, 4-aminobutyrate (GABA), glutathione, uracil, and CMP belonged to the first group, whereas tyrosine, UDP-glucose, leucine, phenylalanine, pyruvate, succinate, glucose 1-phosphate, fumarate, and N-acetyl-*L*-alanine fell in the second group. In contrast, putrescine, GDP, glucose 6-phosphate, valine, and glucose belonged to the third group. The former two metabolites increased and decreased in the *L*-Glu-γ-P_H_ - and α-KG-γ-P_H_ -treated groups, respectively, whereas the opposite was observed for the latter three metabolites.

A further examination *via* heatmap (Fig. S3) of the metabolite levels showed that the three groups formed two major group clusters: the first cluster represented by the control group and *L*-Glu-γ-P_H_ -treated group, whereas the second cluster was only formed by the samples exposed to α-KG-γ-P_H_. This suggested that the effect of α-KG-γ-P_H_ on the *E. coli* intracellular metabolome was somehow more marked than that of *L*-Glu-γ-P_H_.

In conclusion, the results of the multivariate analyses demonstrated that on average the effect of *L*-Glu-γ-P_H_ and α-KG-γ-P_H_ on *E. coli* metabolome was different, with α-KG-γ-P_H_ displaying a greater impact than *L*-Glu-γ-P_H,_ despite the interconversion of these two compounds (see next section).

### α-KG-γ-P_H_ is intracellularly converted to *L*-Glu-γ-P_H_

Since both *L*-Glu-γ-P_H_ and α-KG-γ-P_H_ impacted the *E. coli* metabolism, possibly by entering one or more of the glutamate-dependent metabolic routes (28), the metabolome of the *L*-Glu-γ-P_H_ - and of α-KG-γ-P_H_ -treated groups was further investigated by ^31^P NMR in order to detect and identify possible *H*-phosphinic metabolites originated by the intracellular metabolism of *L*-Glu-γ-P_H_ and α-KG-γ-P_H_. Phosphonate and *H*-phosphinates have a typical chemical shift between 5 to 40 ppm, which is a spectrum zone where only a few phosphorous-containing metabolites appear. This facilitates the detection of the phosphonates and *H*-phosphinates by ^31^P NMR in a mixture of phosphorous-containing metabolites (1). Although the ^31^P NMR spectra revealed the existence of resonance of *H*-phosphinates in both *L*-Glu-γ-P_H_ - and α-KG-γ-P_H_ -treated groups that do not belong to these compounds (Fig. S4), the low signal/noise ratio did not allow their identification. In the case of the cells treated with α-KG-γ-P_H_, the compound itself (α-KG-γ-P_H_) was not found in the extracts, while the major phosphinic compound present was *L*-Glu-γ-P_H_, indicating that *E. coli* can convert the α-KG-γ-P_H_ into *L*-Glu-γ-P_H._ The contrary was not observed, *i.e. L*-Glu-γ-P_H_ is the major phosphinic compound present in *L*-Glu-γ-P_H_-treated cells and α-KG-γ-P_H_ is not detected (Fig. S4).

Thus, the conversion of α-KG-γ-P_H_ into *L*-Glu-γ-P_H_ should be taken into account when interpreting the global effect of the molecules on *E. coli* metabolism.

### The effect of *L*-Glu-γ-P_H_ and α-KG-γ-P_H_ on the *E. coli* proteome

In addition to the identification of the major intracellular metabolites undergoing a significant change in concentration, an analysis was also performed to study how the *L*-Glu-γ-P_H_ or α-KG-γ-P_H_ treatments affected the cells at the proteome level.

**Figure 4:**
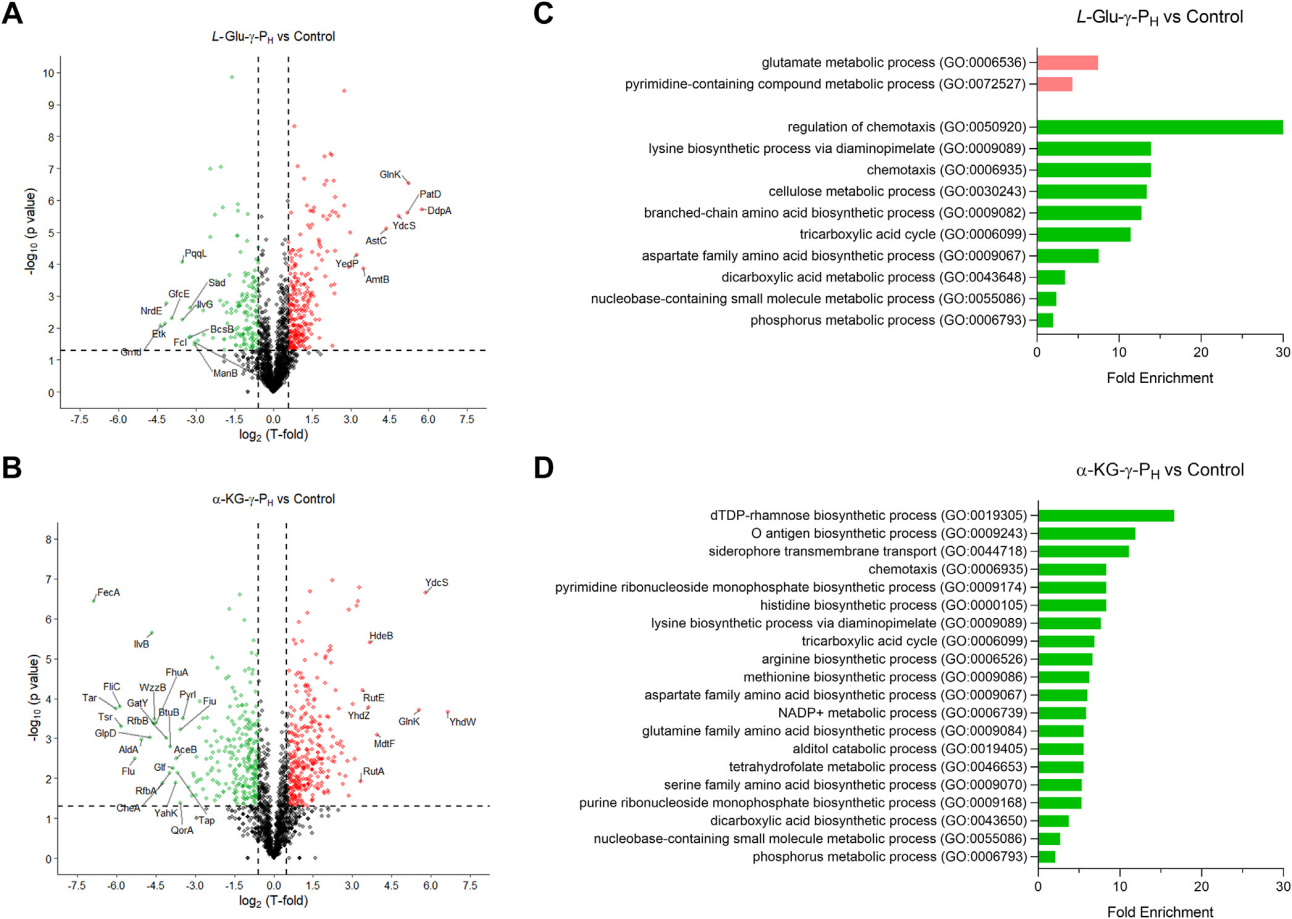
**Volcano plots and Gene Ontology (GO) enrichment analyses.** The graphs illustrate differentially abundant proteins and most affected pathways upon exposure to *L*-Glu-γ-P_H_ or α-KG-γ-P_H_. *A and B*, Volcano plots of all proteins identified in the proteome database when cells were challenged with *L*-Glu-γ-P_H_ (*A*) or α-KG-γ-P_H_ (*B*). Green points: differentially expressed proteins that were significantly downregulated (T-fold < −1.5; *p* < 0.05). Red points: differentially expressed proteins that were significantly upregulated (T-fold > 1,5; *p* < 0.05). Proteins whose T-fold is > 8 or < −8 are text-labelled. *C and D*, Bar charts showing the GO terms for biological process ranked by fold enrichment of cells exposed to *L*-Glu-γ-P_H_ (*C*) or α-KG-γ-P_H_ (*D*). Green bars: significant downregulated pathways (*p* < 0.05). Red bars: significant upregulated pathways (*p* < 0.05).

Based on the metabolomics data clustering, four samples of each of the control and α-KG-γ-P_H_-treated groups were selected for proteome analysis, whereas seven samples of the *L*-Glu-γ-P_H_-treated group were selected, to include both sub-clusters observed in the metabolome (Fig. 3). The shotgun proteomic dataset, comprising 956,723 MS/MS spectra, identified 23,369 unique peptide sequences that allowed monitoring the abundance of 1809 proteins (Table S2). The volcano plots (Fig. 4, *A* and *B*) show in red (increased abundance) and green (decreased abundance) all the proteins that satisfied |T-fold| (TF ≥ 1.5) and *p*-value (≤0.05) (Table S2). The T-fold was used in this study as a parameter combining the fold-change cutoff with a statistical test, as described elsewhere (33). To do so, all numbers <1.0 were converted to their reciprocals and assigned a negative sign.

When comparing each treated group with the control group, differences were observed between the *L*-Glu-γ-P_H_ - and the α-KG-γ-P_H_ -treated groups, with the latter displaying a higher number of affected proteins, *i.e.* 594 in α-KG-γ-P_H_ -treated cells *vs* 423 in *L*-Glu-γ-P_H_ -treated cells. Moreover, the plots show that the overall TF was higher in the α-KG-γ-P_H_ -treated group than in the *L*-Glu-γ-P_H_ -treated group. When all the proteins that satisfied the abovementioned criteria were subjected to Gene Ontology (GO) analysis, the GO terms for biological process revealed that both compounds lead to a decrease in the abundance of proteins involved in the following processes (Fig. 4, *C* and *D*, Table S3): chemotaxis, lysine biosynthesis *via* diaminopimelate, tricarboxylic acid (TCA) cycle, aspartate family amino acid biosynthesis, dicarboxylic acid metabolism, nucleobase-containing small molecule metabolism and phosphorus metabolism. However, α-KG-γ-P_H_ treatment was more markedly affecting the proteome as it caused a change in the abundance of proteins involved in many more processes (Fig. 4*D*), in particular the biosynthesis of methionine, as well as that of polar (Gln, Ser) and positively charged (Arg and His) amino acids, and that of purines and pyrimidines ribonucleoside monophosphates. On the contrary, *L*-Glu-γ-P_H_ treatment uniquely repressed cellulose metabolism and branched-chain amino acids biosynthetic processes (Fig. 4*C*). Strikingly, based on the KEGG analysis, none of the 333 proteins that displayed a significant increase in abundance upon α-KG-γ-P_H_ treatment turned out to hit specific metabolic processes, while in the case of *L*-Glu-γ-P_H_ -treated samples, only a few of the 279 proteins that increased in abundance could be assigned to specific metabolic processes, namely glutamate metabolism and pyrimidine-containing compounds metabolism (Fig. 4*C*).

**Figure 5:**
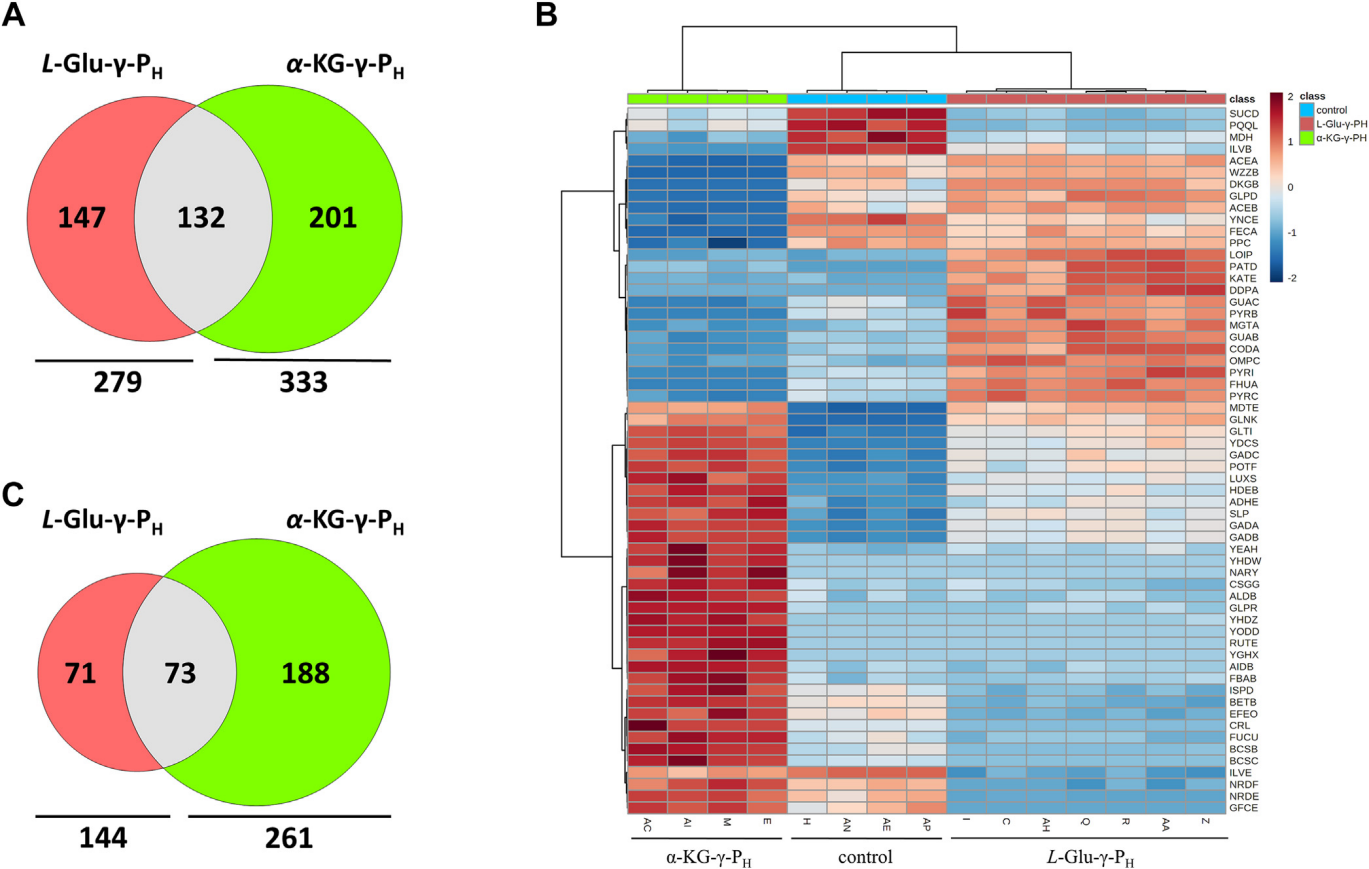
**Vienn diagrams and Heatmap visualization illustrate differentially abundant proteins upon exposure to L-Glu-γ-P**_**H**_**or α-KG-γ-P**_**H**_. *A and B,* values in the Venn diagrams indicate the number of proteins that were upregulated (*A*) or downregulated (*B*) when cells were treated with *L*-Glu-γ-P_H_ or α-KG-γ-P_H._ Numbers in the overlapping circles refer to proteins that were found to be co-upregulated (*A*) or co-downregulated (*B*) by both compounds. *C*, Heatmap visualization for the top 60 proteins based on the analysis of variance (ANOVA) test. Biological replicates (horizontal axis) and single proteins (vertical axis) are separated using a hierarchical clustering based on Euclidean distance.

When proteins with increased abundance in the *L*-Glu-γ-P_H_ -treated group and α-KG-γ-P_H_ -treated group were compared to each other, 132 proteins were found to be in common (Fig. 5*A*; *i.e.* 47% of *L*-Glu-γ-P_H_ -treated group and 40% of α-KG-γ-P_H_ -treated group). This figure goes down to 73 when comparing the protein with decreased abundance in the two groups (Fig. 5*B*; *i.e.* 51% of *L*-Glu-γ-P_H_ -treated group and 28% of α-KG-γ-P_H_ -treated group). The heatmap in Fig. 5*C* compares the top sixty most affected proteins in the three groups. Once again, the extent to which the two compounds affected some cellular components displayed often an opposite trend. In general, α-KG-γ-P_H_ displayed a more pronounced effect, in agreement with the metabolomic results.

### A joint metabolomic and proteomic analysis illustrates the overall impact of the *L*-Glu-γ-P_H_ and α-KG-γ-P_H_ on *E. coli* metabolism

The metabolomics and proteomics results indicated that both *L*-Glu-γ-P_H_ and α-KG-γ-P_H_ significantly affect the *E. coli* metabolism, though to different extent. To better pinpoint the pathways mostly perturbed by *L*-Glu-γ-P_H_ and α-KG-γ-P_H_, an integrative analysis of the proteomics and metabolomics data based on both protein abundances and metabolite concentrations was carried out using the “Joint Pathways Analysis” module from MetaboAnalyst (34). To this aim, the integration of the metabolomic and proteomic data was performed only on the metabolic pathways in which both metabolites and proteins could be matched. The joint analysis was performed using only the metabolites that were significantly affected by *L*-Glu-γ-P_H_ and α-KG-γ-P_H_ compared to the control group according to the univariate statistical analysis. Notably, N-Acetyl-*L*-alanine was excluded from the analysis as very little is known about this metabolite and its synthesis (according to KEGG). Therefore, a total of 25 and 34 statistically significant metabolites for *L*-Glu-γ-P_H_ - and α-KG-γ-P_H_ -treated groups, respectively, were used. Regarding the protein queries, in addition to the proteins that were significantly altered in response to *L*-Glu-γ-P_H_ or α-KG-γ-P_H_ and with a TF > 1.5 and < −1.5 compared to the control group, the proteins with a TF > 1.0 and < −1.0 were also used. In fact, even with a low TF, their upregulation/downregulation could anyhow strengthen the overall impact that *L*-Glu-γ-P_H_ and α-KG-γ-P_H_ have on each metabolic pathway. This increased the total number of proteins used for the joint analysis to 710 and 824 for *L*-Glu-γ-P_H_ - and α-KG-γ-P_H_ -treated group, respectively.

**Figure 6:**
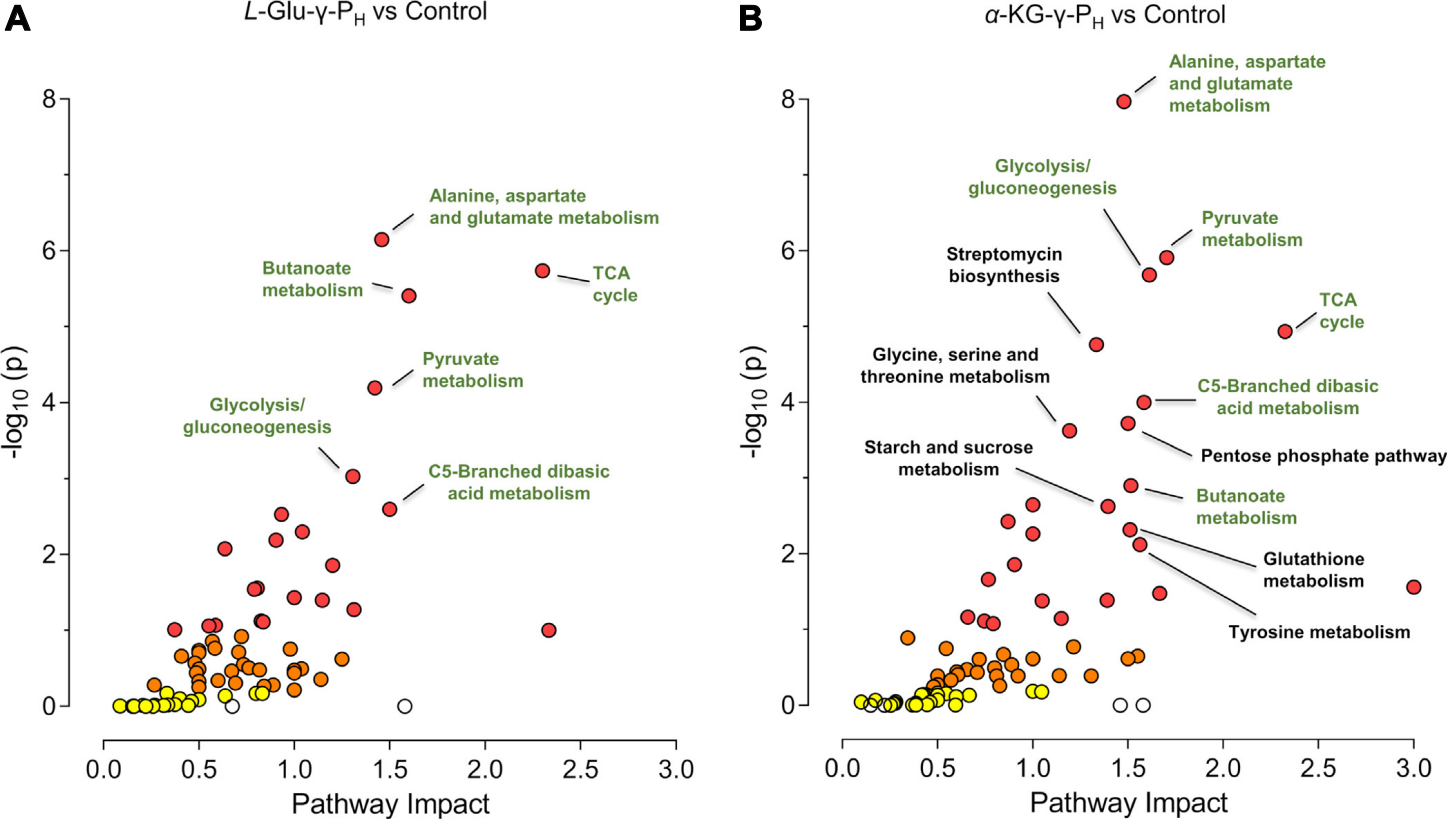
**Integratedprotein-metabolitepathway enrichment analysis.** The integrated protein-metabolite pathway enrichment analysis of cells exposed to L-Glu-γ-P_H_ (*A*) or α-KG-γ-P_H_ (*B*) was performed *versus* the control group. The analysis was carried out using the joint pathway module from MetaboAnalyst (34) in order to integrate the results obtained from the combined proteomics and metabolomics studies performed under the same experimental conditions. The hypergeometric test was used for enrichment analysis, while the degree centrality (which considers the number of links that connect a node) was used for the measure of the topology. Moreover, as an integration method of proteomics and metabolomics data, the combine queries option was used. The database utilized was KEGG. Results were plotted as a function of impact within the pathway and significance. Labeled pathways have a pathway impact index ≥ 1.0 and *p*-value (FDR) < 0.05. Pathways labeled in green are those shared by (*A*) and (*B*). In both graphs, the pathway with the greatest impact was Novobiocin biosynthesis but, due to its low *p*-value, -log_10_(p) < 2, it was not labeled.

Based on the joint analysis, the most affected metabolic pathways were plotted as a function of the level of significance and of the impact that *L*-Glu-γ-P_H_ (Fig. 6*A*) or α-KG-γ-P_H_ (Fig. 6*B*) has on each pathway compared to the control group. Only the metabolic pathways with an impact index ≥ 1.0 and a corrected *p*-value < 0.05 (based on the FDR) were considered significantly affected. The joint analysis shows that *L*-Glu-γ-P_H_ impacted six metabolic pathways (reported as in the KEGG database; Table S4): the TCA cycle, the butanoate metabolism, the C5-branched dibasic acid metabolism, the alanine aspartate and glutamate metabolism, the pyruvate metabolism, and the glycolysis/gluconeogenesis pathway. With the only exception of alanine, aspartate, and glutamate metabolism, which is part of the amino acid metabolism, the other five pathways are all related to carbohydrate metabolism. Of the 25 metabolites used in the analysis, only six of them, *i.e.* citrate, fumarate, succinate, GABA, glutamine, and aspartate, were mapped on the aforementioned pathways. Amongst them, fumarate (TF = −3.1) and succinate (TF = −1.4) contributed to the impact score and the significance of most of these pathways, such as the TCA cycle, butanoate metabolism, alanine aspartate and glutamate metabolism, and pyruvate metabolism. On the other hand, GABA (TF = 2.9) was mapped only on two pathways, *i.e.* butanoate metabolism, and alanine aspartate and glutamate metabolism, whereas citrate (TF = 1.6) was the only metabolite to be uniquely mapped on a single pathway, *i.e.*, the TCA cycle. None of the 25 metabolites could be mapped on the C5-branched dibasic acid metabolism and the glycolysis/gluconeogenesis pathway.

Regarding the α-KG-γ-P_H_ -treated group, in addition to the aforementioned pathways, α-KG-γ-P_H_ significantly impacted the metabolism of tyrosine, glutathione, starch and sucrose, and glycine, serine, and threonine, as well as the pentose phosphate pathway. Furthermore, it also affected the biosynthesis of polyketide sugar unit, which is part of the metabolism of terpenoids and polyketides pathways, as well as the biosynthesis of secondary metabolites, such as streptomycin, and acarbose and validamycin (Fig. 6*B*; Table S5). Overall, the joint analysis revealed that α-KG-γ-P_H_ impacted seven pathways of carbohydrate metabolism, four pathways involved in amino acid metabolism, two pathways related to the biosynthesis of secondary metabolites, and one pathway implicated in the polyketide sugar unit biosynthesis.

A total of 16 out of the 34 metabolites were mapped on almost all the above pathways, except for the acarbose and validamycin biosynthesis and the polyketide sugar unit biosynthesis pathways, for which no matches could be observed. Pyruvate (TF = −1.8) was the most mapped metabolite, being observed in eight pathways, followed by succinate (TF = −1.5) and fumarate (TF = −2.9), which contributed to the impact score and the significance of five and three pathways, respectively (Table S5). Glucose (TF = 3.1), GABA (TF = 4.8), glycine (TF = 1.2) and aspartate (TF = 10.0) were each mapped on two metabolic pathways, whereas lactate (TF = −1.5), ethanol (TF = −1.2), glutathione (TF = 4.78), putrescine (TF = −2.1), glucose 6-phosphate (TF = 2.5), glycogen (TF = 1.5), choline (TF = −1.4) and threonine (TF = −2.1) were each uniquely mapped on a single pathway (Table S5). This latter result, in agreement with the proteomic data, explains why the total number α-KG-γ-P_H_ -affected pathways, as revealed by the joint analysis, was higher than in the *L*-Glu-γ-P_H_ -treated cells.

Therefore, the joint analysis confirmed the greater pervasiveness of α-KG-γ-P_H_ than *L*-Glu-γ-P_H_ on *E. coli* metabolism. Alike *L*-Glu-γ-P_H_, though to a greater extent, α-KG-γ-P_H_ significantly perturbed the central carbon metabolism and the biosynthesis of some amino acids.

### Exposure to *L*-Glu-γ-P_H_ and α-KG-γ-P_H_ leads to the upregulation of proteins involved in acid resistance and nitrogen assimilation and metabolism

Despite in KEGG and GO the stress responses pathways are not automatically pinpointed, the metabolomic analysis clearly indicated that some metabolites (*i.e.* glutamate, GABA, and glutamine) that participate in the glutamine/glutamate-dependent acid resistance (AR2) in *E. coli* and other bacteria (27, 35, 36) are increased in both *L*-Glu-γ-P_H_ - and α-KG-γ-P_H_ -treated groups (Table S1). The activation of the acid stress response was confirmed by the finding that the enzymes and structural proteins that participate in AR2 are indeed amongst the top overexpressed proteins in the proteomic analysis in both *L*-Glu-γ-P_H_ - and α-KG-γ-P_H_ -treated groups as compared to the control (Table S2). Specifically, these proteins include the ones that are listed to follow, together with the TF provided in parenthesis. The two glutamate decarboxylase isoforms GadA (TF = 4.83 for *L*-Glu-γ-P_H_ -treated group and 9.71 for α-KG-γ-P_H_ -treated group) and GadB (TF = 4.65 and 9.40), the glutamate/GABA antiporter GadC (TF = 5.02 and 9.05), the outer membrane protein Slp (TF = 2.37 and 4.00), the acid resistance membrane protein HdeD (TF = 5.71 and 5.75), which protect from acidic metabolites, the periplasmic chaperones HdeA (TF = 5.23 and 6.08) and HdeB (TF = 5.32 and 12.61), which confer protection from acid-denatured proteins, and the two multidrug resistance transporter MdtE (TF = 6.66 and 7.38) and MdtF (TF = 7.86 and 15.50), which together with TolC form the tripartite efflux system MdtEF-TolC. Notably, with the exception of GadB, GadC, and GlsA1 (TF = 2.05 and 2.84), all the genes encoding the above-mentioned proteins are located in a genomic island named AFI (Acid Fitness Island (37, 38). The AR2 and AFI genes are under the control of specific transcriptional regulators, *i.e.* GadX and GadY, also encoded in the AFI (39) and shown to be involved in the repression of BtuB, as observed in this study (see discussion).

Finally, the in-depth analysis of the proteomic data revealed that up to 38 and 31 proteins, whose genes are known or predicted to be activated in nitrogen starvation (40, 41, 42), were upregulated (TF ≥ 1.5) in response to *L*-Glu-γ-P_H_ and α-KG-γ-P_H_, respectively (Table S2). Most of these genes are part of the transcriptional circuit of the nitrogen regulator I (NRI, also known as NtrC or σ^54^). NRI is a member of the two-component regulatory system NRII/NRI, which controls the expression of the nitrogen-regulated (*ntr*) genes in response to nitrogen limitation (40, 41, 42). Indeed, a sharp increase in the levels of key proteins involved in nitrogen assimilation was observed, such as NRI (TF = 2.91 and 2.69), NRII (TF = 2.80 and 2.40), the ammonia channel AmtB (TF = 11.14 and 8.50), glutamine synthetase GlnA (TF = 3.56 and 2.92), and the nitrogen regulatory protein P-II GlnK (TF = 37.62 and 46.83) (Table S2).

**Figure 7:**
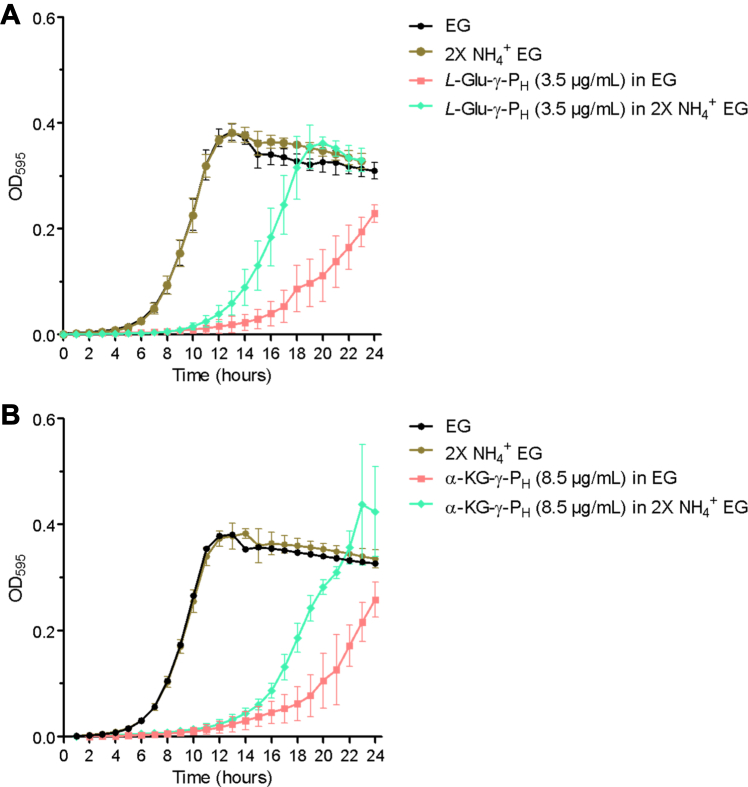
**The increment in NH**_**4**_^**+**^**in EG medium improves the fitness of the cells when exposed to *L*-Glu-γ-P**_**H**_**and α-KG-γ-P**_**H**_**.** At sub-inhibitory concentrations, *L*-Glu-γ-P_H_ (3,5 μg/ml; *A*) and α-KG-γ-P_H_ (8.5 μg/ml, *B*) delay cell growth in EG medium pH 7.0 (*red* squares) compared to the untreated group (*black* dots). In the same experimental conditions, a 2-fold increase in the ammonium content (2X NH_4_^+^ EG) mitigates the inhibitory effect of *L*-Glu-γ-P_H_ (*A*) and α-KG-γ-P_H_ (*B*) on cell growth (*green* rhombus) compared to the untreated group (*brown* dots). No significant NH_4_^+^-related effect is observed if the untreated cells are grown in 2× NH_4_^+^ EG (*brown* dots) compared to EG (*black* dots). Data, shown as mean ± sd, are the averages of at least three independent experiments.

Therefore, it was hypothesized that, as a result of their metabolic impact, *L*-Glu-γ-P_H_ and α-KG-γ-P_H_ may deplete cells of nitrogen. This would lead to a nitrogen starvation response, which would stimulate the expression of the *ntr* genes *via* NRI in an attempt to provide cells with nitrogen through different sources. In agreement with this hypothesis, when cells were grown in EG medium, pH 7, with the same sub-inhibitory concentrations of *L*-Glu-γ-P_H_ and α-KG-γ-P_H_ used for the metabolomics/proteomics experiments, *that is,* 3.5 and 8.5 μg/ml, respectively, a 2-fold increase in the ammonium content (∼32 mM), which is the sole N-source in EG medium, mitigated the inhibitory activity of *L*-Glu-γ-P_H_ (Fig. 7*A*) and α-KG-γ-P_H_ (Fig. 7*B*). Notably, the increase in the ammonium content did not have any effect *per se* on cell growth in the untreated control (Fig. 7, *A* and *B*). Therefore, it was concluded that the increment in nitrogen availability improved the fitness of the cells when challenged with *L*-Glu-γ-P_H_ or α-KG-γ-P_H_, but it did not abolish the inhibition at the tested concentrations.

## Discussion

Due to the spread at an alarming rate of “superbugs”, *i.e.*, pathogenic bacteria resistant to more than one antibiotic, new therapeutic options are needed (29, 43), (https://www.who.int/docs/default-source/documents/no-time-to-wait-securing-the-future-from-drug-resistant-infections-en.pdf). Phosphonates, phosphinates and *H*-phosphinates, *i.e.*, natural products containing one (or two) carbon-phosphorus (C-P) bond(s) or containing phosphorus-hydrogen (P-H) bond in addition to P-C bond, could be exploited as potential antibiotics. In fact, the C-P bond(s) and P-H make phosphonates, phosphinates, and *H*-phosphinates bioactive molecules that could be enzymatically metabolized, producing novel biologically active phosphorus-containing molecules that act as enzyme inhibitors (4, 44). In this light, the phosphonic, phosphinic, and *H*-phosphinic analogs of natural amino acids have drawn particular interest. In this regard, the antimicrobial activity of the phosphonic and *H*-phosphinic analogs of *L*-alanine (*i.e.* 1-aminoethylphosphonic and 1-aminoethylphosphinic acids, respectively) is well-known (45, 46, 47, 48, 49, 50, 51). Moreover, the dipeptide (*L*-leucyl-*L*-phosphinoithricin) and tripeptide (Bialaphos) derivatives of *L*-PT, *i.e.*, the phosphinic analog of glutamate, were recently found to be effective on multidrug-resistant (MDR) clinical isolates of *K. pneumoniae*, resistant to more than 20 commercially available antibiotics (11).

*L*-Glu-γ-P_H_, the *H*-phosphinic analog of glutamate, is a metabolic intermediate precursor occurring during the biosynthesis of Bialaphos in *S. hygroscopicus* and *S. viridochromogenes*, with a MIC_90_ of 7 μg/ml (42 μM) when assayed on the *E. coli* K12 strain MG1655 (20, 21, 22, 26). Owing to the *H*-phosphinic group acting as a bioisostere of the carboxyl group, *L*-Glu-γ-P_H_ can be metabolized by glutamate-dependent enzymes with the production of *L*-Glu-γ-P_H_ -derived *H*-phosphinic metabolites, such as GABA-P_H_ and succinate-P_H_ (26). This is of relevance given the role of glutamate in microbial metabolism. In fact, in bacteria glutamate plays an important role not only as a proteinogenic amino acid but also as a key metabolite for many metabolic pathways, some of which are essential for cell survival. These pathways include the biosynthesis of amino acids, purine and pyrimidines, glutathione, and some cofactors as well as nitrogen metabolism (25, 28). In support of this, in *E. coli* glutamate is one of the most abundant intracellular metabolites, and the main counter-ion of potassium (52). In addition, in many enteropathogenic bacteria, including pathogenic *E. coli* strains, glutamate is also an essential determinant of survival at low pH (24, 27).

In this work, the metabolic impact of *L*-Glu-γ-P_H_ and its keto-acid derivate α-KG-γ-P_H_, also exhibiting inhibitory activity though to a lesser extent (MIC_90_ of 13 μg/ml, 78 μM), was investigated at the metabolic and proteomic level through an in-depth multi-omics approach, to provide insights on their mechanism of action, as well as on the molecular responses to these two compounds that eventually allow the cell to withstand their inhibitory activity. Our analysis provides also insight into the adaptation of the *E. coli* metabolism to the *H*-phosphinic compounds at subinhibitory concentrations. This topic is of interest because exposure at subinhibitory concentrations of antibiotics has been reported to promote antimicrobial resistance traits (53, 54, 55). It is therefore crucial to understand what are the metabolic adaptations that lead to the development of resistance mechanisms.

To the best of our knowledge, this work is the first combined metabolomic and proteomic study on the effect of phosphinic compounds on bacterial metabolism.

### Impact of *L*-Glu-γ-P_H_ and α-KG-γ-P_H_ on the *E. coli* metabolome and proteome

The ^1^H NMR metabolomic analysis, corroborated by the multivariate statistical analyses (PCA, PLS-DA, and heatmap), showed that the α-KG-γ-P_H_ -treated group displayed overall greater differences in the metabolomic profile than the *L*-Glu-γ-P_H_ -treated cells (Fig. 3 and Table S1). Interestingly, a detailed analysis of the ^1^H NMR spectra revealed the presence of peaks characteristic of *L*-Glu-γ-P_H_ in the spectra of the α-KG-γ-P_H_ -treated group, whereas the opposite was not observed (Fig. S4). This finding provides a strong indication that α-KG-γ-P_H_ can be intracellularly transformed into *L*-Glu-γ-P_H_, but the opposite does not happen. Therefore, the deeper impact of α-KG-γ-P_H_ on *E. coli* metabolome may be explained assuming that α-KG-γ-P_H_ acts both as such and in the form of *L*-Glu-γ-P_H_. Despite this, α-KG-γ-P_H_ has a MIC_90_ of 13 μg/ml (78 μM), under the assay condition used in this study, *i.e.*, almost twice as high as *L*-Glu-γ-P_H_ (7 μg/ml, 42 μM) (26). These differences may be attributed to the different uptake systems. In fact, assuming that *L*-Glu-γ-P_H_ and α-KG-γ-P_H_ are uptaken through the import machinery of glutamate and α-ketoglutarate, to date only one α-ketoglutarate permease (KgtP) has been found in *E. coli* (56), whereas glutamate transport can be performed by up to four transporters: the glutamate/GABA antiporter GadC; the sodium-dependent glutamate transporter GltS (also known as GltC); GltP, a proton-dependent transporter for glutamate and aspartate, and the glutamate/aspartate ATP-binding cassette (ABC) transporter GltIJKL (57). Interestingly, in addition to GadC, the periplasmic-binding protein GltI and the ATP-binding protein GltL, both subunits of the ABC transporter GltIJKL (58, 59, 60, 61), in this study were found to be significantly upregulated in response to the exposure to *L*-Glu-γ-P_H_ and α-KG-γ-P_H_. In contrast, the intracellular levels of α-ketoglutarate permease KgtP were not affected. Therefore, α-KG-γ-P_H_ could be subjected to a more limited import rate than *L*-Glu-γ-P_H_, which might provide a possible explanation for its MIC_90_.

The analysis of the proteome upon exposure to either *L*-Glu-γ-P_H_ or α-KG-γ-P_H_ confirmed the higher perturbation caused by the latter molecule on *E. coli*. Indeed, *L*-Glu-γ-P_H_ significantly impacted the abundance of 423 proteins, of which 279 and 144 were up- and downregulated, respectively. In contrast, 594 proteins were significantly affected by α-KG-γ-P_H_, of which 333 and 261 were up- and downregulated, respectively (Fig. 5, Table S2). When a GO analysis on these proteins was performed, it turned out that both compounds significantly led to a decrease in the abundance of proteins involved in metabolic processes where glutamate is a key metabolite serving either as a nitrogen donor or as a building block (62). These processes include the biosynthesis of branched-chain amino acids and aspartate (Fig. 4, *C* and *D*, Table S3). Furthermore, metabolic processes related to the central carbon metabolism, such as the dicarboxylic acid metabolic process, TCA cycle, and cellulose metabolic process (the latter only for *L*-Glu-γ-P_H_) were also negatively impacted as indicated by the downregulation of many of the enzymes involved in these pathways. This finding may explain the bacteriostatic effect that *L*-Glu-γ-P_H_ and α-KG-γ-P_H_ have on cell growth. Notably, the exposure to both *L*-Glu-γ-P_H_ and α-KG-γ-P_H_ severely affected the bacterial chemotaxis, with most of the proteins involved being sharply down-regulated (hereby, values refer to *L*-Glu-γ-P_H_ and α-KG-γ-P_H_, respectively in Table S2), such as CheA (TF = −6.46 and −19.20), CheB (TF = −2.19 and −2.50), CheY (TF = −3.75 for both), and CheZ (TF = −2.75 and 8.25). In this regard, the chemoreceptors Tsr (TF = −5.44 and −56.75), Tar (TF = −4.81 and −65.25), and Tap (TF = −3.43 and −12.75), all responding to amino acid stimuli, along with the flagellin FliC (TF = −59.25 but only for α-KG-γ-P_H_) were among the most downregulated proteins observed in this study [(63) and references therein]. This may suggest that part of the growth inhibition is due to the reduced “hunt” for amino acids. The GO analysis confirmed that the α-KG-γ-P_H_ treatment had a more pronounced effect on *E. coli* metabolism by repressing additional processes not observed with *L*-Glu-γ-P_H_, such as the biosynthesis of glutamine, serine, arginine, and tetrahydrofolate, probably as a result of *H*-phosphinic derivatives originated by the intracellular metabolism of α-KG-γ-P_H_ (Fig. 4*D*, Table S3). Additionally, an effect on the biosynthesis of methionine and lysine as well as on metabolic processes involving purines and pyrimidines was also observed. The negative impact on the siderophore transmembrane transport, only observed with α-KG-γ-P_H_, may find explanation by the sharp downregulation of the vitamin B_12_ (cobalamin) receptor BtuB (TF = −15.50), the ferrichrome outer membrane transporter FhuA (TF = −22.50), and the Fe(3^+^) dicitrate transport protein FecA (TF = −118.50), which are all TonB-dependent transporters involved in the uptake and transport of cobalamin and iron (64, 65, 66). BtuB is also known to be implicated in the import of colicins, along with the porin OmpF (TF = −3.51) (67). Notably, BtuB, FhuA, and FecA were amongst the most downregulated proteins in response to α-KG-γ-P_H_. Possible involvement of GadX and GadY, transcriptional activators of the AR2 genes, amongst the most upregulated genes in this study, can be the likely explanation in accordance with the literature (68).

Interestingly, unlike *L*-Glu-γ-P_H_, the treatment with α-KG-γ-P_H_ turned out to also negatively impact the bacterial outer membrane biogenesis by downregulating enzymatic activities involved in the biosynthesis of the LPS O-antigen (Table S2), such as the dTDP-glucose 4,6-dehydratase 1 enzyme RfbB (TF = −17), the dTDP-4-dehydrorhamnose reductase protein RfbD (FC = −6), the glucose 1-phosphate thymidylyltransferase 1 RfbA (TF = −15.75), and the dTDP-4-dehydrorhamnose 3,5-epimerase RfbC (TF = −2.25), which are all part of the *rfb* gene cluster (69, 70). Moreover, though not highlighted by the GO analysis, it was found that α-KG-γ-P_H_ had a strong negative effect on the cellular levels of the enzymes UDP-galactopyranose mutase GlF (TF = −14.50), β-1,6-galactofuranosyltransferase WbbI (TF = −8.75), and of the putative lipopolysaccharide biosynthesis O-acetyl transferase WbbJ (TF = −2.5), also involved in the LPS biosynthesis (71).

Hence, though both molecules negatively impacted the roots of the central carbon metabolism and glutamate-related amino acids, α-KG-γ-P_H_ stood out for its greater effect on *E. coli* metabolism by also sharply perturbing pathways related to outer membrane homeostasis and the uptake of nutrients essential for cell viability, such as cobalamin and iron.

### The overall impact of *L*-Glu-γ-P_H_ and α-KG-γ-P_H_ on *E. coli* metabolism

To pinpoint the pathways primarily perturbed by *L*-Glu-γ-P_H_ and α-KG-γ-P_H_, an integrative analysis of the proteomic and metabolomic data was performed. Only pathways with impact score > 1 and corrected *p*-value (FDR) < 0.05 were considered significantly perturbed (Tables S4 and S5). The joint analysis revealed that *L*-Glu-γ-P_H_ impacted five metabolic pathways related to carbohydrate metabolisms, such as the TCA cycle, pyruvate metabolism, glycolysis/gluconeogenesis, butanoate metabolism, and C5-branched dibasic acid metabolism, whereas only one pathway of the amino acid metabolism, *i.e.*, alanine, aspartate, and glutamate metabolism, was found significantly affected. As for α-KG-γ-P_H_, six additional pathways were significantly perturbed: two were related to carbohydrate metabolism (*i.e.* starch and sucrose metabolism, and pentose phosphate pathway); three to amino acid metabolism (*i.e.* glycine, serine, and threonine metabolism, glutathione metabolism, and tyrosine metabolism); one additional pathway related to the biosynthesis of secondary metabolites.

In both *L*-Glu-γ-P_H_ - and α-KG-γ-P_H_ -treated groups, the alanine, aspartate, and glutamate metabolism stood out as one of the most affected metabolic roots (Fig. 6, *A* and *B*, Tables S4 and S5). This pathway represents a key node in microbial metabolism as it provides metabolic precursors for the biosynthesis of amino acids, such as histidine, arginine, and proline, as well as of pyrimidines and purines, amino sugars and nucleotide sugars (25, 62). Furthermore, the above metabolism is strongly intertwined with pathways related to the central carbon metabolism, such as the TCA cycle, glycolysis/gluconeogenesis, and pentose phosphate pathway (though the latter only by α-KG-γ-P_H_), which in fact turned out to be significantly affected. As a result, most of the observed “omic” changes in the *L*-Glu-γ-P_H_ - and α-KG-γ-P_H_ -treated cells may be attributed to their impact on the alanine, aspartate, and glutamate metabolism.

The analysis revealed that aspartate, GABA, glutamine, citrate, fumarate, and succinate were the only metabolites (out of 25) to be significantly mapped on the aforementioned pathways in *L*-Glu-γ-P_H_ -treated cells (Table S4). Their observed TFs may be attributed to the up- and downregulation of several enzymatic activities. For instance, the increase in aspartate may be the result of the lower abundance of the aspartate–ammonia ligase AsnA (TF = −4.18), the upregulation of the isoaspartyl peptidase IaaA (TF = 2.24), which exhibits asparaginase activity, the downregulation of the aspartate oxidase NadB (TF = −4.64), which catalyzes the oxidation of *L*-aspartate to iminoaspartate (first step in the *de novo* biosynthesis of NAD^+^). Notably, these three enzymes are part of the alanine, aspartate, and glutamate metabolism. Additional contributions may derive from the overall deregulation of the *L*-lysine biosynthesis, and of *L*-cysteine and *L*-methionine metabolisms, as indicated by the downregulation of several enzymes that map in these pathways. As for glutamine, its increase may be attributed to the upregulation of GS (TF = 3.56). In parallel, increased enzymatic activity of some of the enzymes of the GABA-shunt pathway, such as GadA and GadB (TF = 4.83 and 4.65, respectively), which decarboxylate glutamate yielding GABA, as well as of the γ-aminobutyraldehyde dehydrogenase PatD (TF = 36.43), which oxidizes γ-aminobutyraldehyde to GABA in the *L*-arginine and *L*-ornithine degradation pathway, may lead to the observed accumulation of GABA.

Finally, the observed decrease in succinate and fumarate is likely the result of a perturbation of the central carbon metabolism, which is evident by the marked downregulation of numerous enzymes involved in the TCA cycle and pyruvate metabolism. Together with this, the slight upregulation of enzymes in the glycolysis/gluconeogenesis route may explain the increase in citrate levels. It is reasonable to claim that these findings are likely the result of an adaptive response rather than a direct inhibition of any enzymes in these pathways by *L*-Glu-γ-P_H_.

As already mentioned, α-KG-γ-P_H_ exhibited a greater pervasiveness than *L*-Glu-γ-P_H_ on *E. coli* metabolism, which was reflected by the higher number of metabolites (16 out of 34) and proteins significantly up- and downregulated (594 out of 812) that were mapped onto 14 *E. coli* pathways (Table S5). Nevertheless, likely because of its intracellular conversion to *L*-Glu-γ-P_H_, to some extent the impact of α-KG-γ-P_H_ on *E. coli* metabolism was similar to *L*-Glu-γ-P_H_. For example, alike *L*-Glu-γ-P_H,_ the sharp increase in aspartate could be attributed to the downregulation of AsnA (TF = −7.17), along with additional enzymes uniquely impacted by α-KG-γ-P_H_, such as the aspartate carbamoyltransferase complex subunits PyrB and PyrI (TF = −5.24 and −11.10, respectively), which catalyzes the first step in the *de novo* pyrimidine biosynthesis pathway, and the NADP^+^-specific glutamate dehydrogenase GdhA (TF = −3.04), which catalyzes the reversible oxidative deamination of glutamate to α-ketoglutarate and ammonia. This latter reaction represents a key metabolic link between the TCA cycle and nitrogen assimilation (9, 72). As observed in *L*-Glu-γ-P_H_ -treated cells, additional contribution may derive from the repression of the *L*-lysine biosynthesis and of *L*-cysteine and *L*-methionine metabolism, as well as of glycine, serine and threonine metabolism, *i.e.* pathways that deplete the aspartate pool to synthesize aspartate-derived amino acids. This is in agreement with the reduction of threonine and putrescine levels observed by ^1^H NMR (Table S1). Alike in *L*-Glu-γ-P_H_ -treated cells, the sharp upregulation of GadA and GadB (TF = 9.71 and 9.40, respectively) and of PatD (TF = 6.75) would account for the increment in GABA levels (Table S1). Finally, the observed decrease in pyruvate, succinate and fumarate would reflect the perturbation of the central carbon metabolism, similarly to what was observed with *L*-Glu-γ-P_H_ (Table S1).

Although the level of significance was above the threshold (adjusted *p*-value > 0.05), the exposure to *L*-Glu-γ-P_H_ and α-KG-γ-P_H_ may attenuate to some extent the valine, leucine, and isoleucine biosynthetic pathway, as suggested by the observed downregulation of many of the enzymes of this pathway (Tables S4 and S5). The *L*-threonine dehydratase biosynthetic IlvA (TF = −3.78) and the branched-chain-amino-acid aminotransferase IlvE (TF = −3.04) were the most negatively impacted enzymes in *L*-Glu-γ-P_H_ -treated cells; whereas the acetolactate synthase isozyme one large and small subunits IlvB and IlvN (TF = −25.20 and −2.25, respectively), the ketol-acid reductoisomerase (NADP^+^-dependent) IlvC (TF = −2.84), and the two-isopropyl malate synthase LeuA (TF = −3.50) were downregulated upon exposure to α-KG-γ-P_H_. These findings are in agreement with the observed decrease in the levels of valine, leucine, and isoleucine (Table S1).

Notably, the exposure to both molecules led to an increase in the levels of intracellular glutathione (Table S1). Nevertheless, the joint analysis was only able to link this metabolite to its biosynthetic pathway without identifying any significant perturbation that could explain the increment in glutathione levels.

### Activation of the glutamate-dependent acid resistance system

A notable finding of this work was that both compounds caused a significant increase in the expression of proteins encoded by the genes located in the AFI (Acid Fitness Island) (37, 38), such as Slp, HdeA, HdeB, HdeD, MtdE, MtdF, GadA, as well as the glutaminase GlsA1, active under acidic conditions (73), GadB (the other isoform of glutamate decarboxylase in *E. coli*, together with GadA) and GadC, the glutamine/glutamate-GABA antiporter. All the above proteins, and particularly the latter four, are of key importance in conferring protection from environmental acid stress as demonstrated in several studies and also shown in STRING (https://string-db.org/network/511145.b3508). Given that the experimental conditions in this work do not use an acidic growth medium and the bacterial growth was halted at OD_600_ of 1.0, *i.e.* when typically the AR2 is repressed [*i.e.* exponential phase, pH 7.0, (74)], the likely explanation is that, upon entry of *L*-Glu-γ-P_H_ or α-KG-γ-P_H_, intracellular acidification occurs, which the cell compensates by activating the relevant genes. This finding would fit with the experimental setup that primarily aims at observing the adaptation response to sublethal concentrations of *L*-Glu-γ-P_H_ or α-KG-γ-P_H_. Preliminary experiments indicate that in the *E. coli* triple mutant Δ*gadABC* the MIC_90_ for *L*-Glu-γ-P_H_ increases to 11 μg/ml (66 μM), implying that activation of AR2 in the absence of extracellular acidification conditions may exacerbate the outcome, rendering bacteria more susceptible to the drug rather than conferring a protective effect. Further experiments will be needed to better understand this observation, which would be in line with the well-known finding that a decrease in intracellular pH favors antibiotic resistance (75, 76, 77).

### Ammonia mitigates the inhibitory activity of *L*-Glu-γ-P_H_ and α-KG-γ-P_H_

The analysis of the proteome showed that, though with some differences, the exposure to *L*-Glu-γ-P_H_ and α-KG-γ-P_H_ was accompanied by the upregulation of proteins known to be involved in nitrogen assimilation and metabolism (40, 41, 42). It should be recalled that when the cell is in an N-limited state, as indicated by a decrease in the glutamine/α-ketoglutarate ratio, the transmitter protein NRII activates itself by autophosphorylation, and then it transfers the phosphate group to NRI. Once phosphorylated, NRI-P binds directly to DNA and activates the expression of the *ntr* genes. The latter genes encode proteins, identified as more abundant in our treated samples, directly or indirectly involved in the uptake/transport of glutamate, aspartate, glutamine, ammonium, polyamines, di- or oligopeptides, as wells in the catabolism of amino acids or nucleobases as N-sources. In addition to NRI, it was observed the upregulation of proteins encoded by genes that are transcriptionally activated by Nac, another nitrogen transcriptional regulator (Table S2) (9, 72). This suggested that the compounds under study may cause cellular depletion of nitrogen, leading to a nitrogen starvation response that stimulates the expression of nitrogen-regulated (*ntr*) genes *via* NRI. This hypothesis was supported by the observation that increasing the ammonium content mitigated the inhibitory activity of both *L*-Glu-γ-P_H_ and α-KG-γ-P_H_, while having no substantial effects on the untreated cells (Fig. 7). Notably, the observed mitigation effect on the α-KG-γ-P_H_ -treated cells was slightly less than *L*-Glu-γ-P_H_, which may find explanation in the broader metabolic impact that α-KG-γ-P_H_ has on *E. coli* metabolism.

It is well-known that in bacteria nitrogen assimilation leads to the fixation of ammonium in the form of aspartate, glutamate, and glutamine, which are key nitrogen donors or act as precursors of the majority of nitrogen-containing molecules in the cell (9, 72). In agreement, the alanine, aspartate, and glutamate metabolism (where aspartate, glutamate, and glutamine are central metabolites) turned out to be one of the most perturbed pathways in response to *L*-Glu-γ-P_H_ and α-KG-γ-P_H_, as indicated by the joint pathway analysis. Taking all of this into account, it is plausible to hypothesize that the exposure to *L*-Glu-γ-P_H_ and α-KG-γ-P_H_ interferes with the ammonia fixation into bioorganic molecules, causing growth arrest, thus explaining at least in part the bacteriostatic effect, and forcing the cell to reorganize its metabolism with concomitant downregulation of the nitrogen-depleting pathways from one side, and increased expression of proteins involved in nitrogen-assimilation routes to the other. Given the well-known inhibitory effect that Bialaphos (in the form of *L*-PT) has on GS in plants (7, 8), which plays a central role in nitrogen assimilation (9), it can be hypothesized that *L*-Glu-γ-P_H_ and α-KG-γ-P_H_ (the latter molecule upon its intracellular conversion to *L*-Glu-γ-P_H_) could also be inhibitors of glutamine synthetase (GS), though a different mechanism might be involved. This finds support in the observed upregulation of GS (Table S2), which may be interpreted as a responsive mechanism enabling cells to withstand the inhibitory effect of *L*-Glu-γ-P_H_ on GS. In this regard, further insights may come by studying *in vitro* and *in vivo* the inhibitory activity of *L*-Glu-γ-P_H_ and α-KG-γ-P_H_ in GS-depleted cells, as well as in cells depleted of other enzymes that are part of the nitrogen-starvation response. While this accounts for many of the proteomic and metabolic results, additional effects on the cellular metabolism unrelated to nitrogen assimilation should not be ruled out. Although nitrogen depletion was one of the phenotypic changes revealed and investigated in this work, *L*-Glu-γ-P_H_ and α-KG-γ-P_H_ treatment target other *E. coli* metabolic traits. Indeed, our study provides evidence that the studied molecules very likely hit more than one target. Multi-target antibiotics are currently considered amongst the most promising antibiotics because, as compared to drugs that selectively act on a single target, are less at risk of losing effectiveness by single mutations or changes in expression, ultimately leading to the fast appearance of antibiotic resistance traits that globally are accumulating at a worrisome rate (29).

In conclusion, this work sheds light on the potential of the glutamate *H-*phosphinic analogue *L*-Glu-γ-P_H_ and of its ketoacid derivate, α-KG-γ-P_H_, as antimicrobial agents and provides insights into their effects on *E. coli* metabolism by a combined multi-omics approach. Despite the possibility of α-KG-γ-P_H_ and *L*-Glu-γ-P_H_ interconversion in *E. coli*, *L*-Glu-γ-P_H_ and α-KG-γ-P_H_ have different effects on *E. coli* metabolome and proteome, with α-KG-γ-P_H_ showing a greater impact than *L*-Glu-γ-P_H_, In fact, while sharing some of the perturbed metabolic pathways, likely owing to the enzymatic conversion of α-KG-γ-P_H_ to *L*-Glu-γ-P_H_, others were uniquely affected by each compound. For instance, it was found that α-KG-γ-P_H_, but not *L*-Glu-γ-P_H_, downregulated several proteins involved in the uptake of cobalamin and iron, as well as in LPS O-antigen synthesis. Nevertheless, in both cases, an overall downregulation of the aspartate-dependent pathways, as well as of the central carbon metabolism, was observed. Additionally, this work shows that the exposure to *L*-Glu-γ-P_H_ and α-KG-γ-P_H_ causes an activation of the acid stress response and likely starve cells of nitrogen. Altogether, these findings may account for the observed bacteriostatic effect of these two compounds. Further studies could explore the potential applications of these compounds as antibacterial agents and investigate their effects on other bacterial species. Additionally, understanding the enzymatic metabolism of *L*-Glu-γ-P_H_ and α-KG-γ-P_H_ could offer novel insights into bacterial metabolism and open to the exploitation of these molecules as antibiotics.

## Experimental procedures

### Materials

Ingredients for Lysogeny Broth (LB) medium were from BD-Difco (Becton, Dickinson and Company). Sodium chloride, ammonium chloride and *D*-glucose were from Carlo Erba Reagents. Ingredients for E medium (78) were purchased from VWR International and AppliChem.

Deuterium oxide (D_2_O) containing 3-(trimethylsilyl)-2,2,3,3-tetradeuteropropionic acid (TSP) and methanol were from VWR International and Sigma-Aldrich, respectively. Pyridoxal hydrochloride was from Calbiochem, AlCl_3_·6H_2_O was from Acros.

Ion-exchange chromatography was carried out on Dowex 50W × 8, H^+^-form, 100 to 200 mesh (BioRad, USA). TLC was carried out on Kieselgel 60 F^254^ plates (Merck, Germany).

### Synthesis and analysis of phosphinic compounds

*L*-Glu-γ-P_H_ was synthesized as described elsewhere (26). The powder was dissolved in Milli-Q water to obtain a solution, which was adjusted to pH 7.0 by dropwise addition of 5 M NaOH. The stock solution (2.8 mg/ml; ∼17 mM) was filter-sterilized, dispensed into aliquots, and stored at −20 °C.

To synthesize α-KG-γ-P_H,_ *rac*-Glu-γ−P_H_, prepared as described elsewhere (79), was used as starting material. A solution of *rac*-Glu-γ−P_H_ (240 mg, 1.44 mmol), pyridoxal hydrochloride (422 mg, 2.07 mmol), and AlCl_3_·6H_2_O (50 mg, 0.21 mmol) in water (100 ml) was adjusted to pH 6.7 with aq. NH_4_OH and refluxed in argon atmosphere for 90 min. The reaction mixture was concentrated *in vacuo* to ∼ 6 ml and α-KG-γ-P_H_ was isolated by column chromatography on Dowex 50W × 8 column (100–200 mesh, H^+^-form, V = 40 ml) eluting with water. Acidic fractions were analyzed by ^31^P NMR and those containing target *H-*phosphinate were combined and evaporated to dryness *in vacuo*. The residue was co-evaporated *in vacuo* with water (3 × 5 ml) and dried *in vacuo* over P_2_O_5_/KOH to give α-KG-γ-P_H_ (80 mg, *i.e.* 34% yield) as a clear glass-like semisolid oil.

α-KG-γ-P_H_ was dissolved in Milli-Q water and the solution was adjusted to pH 7.0 with 5 M NaOH. The stock solution (4 mg/ml; ∼ 24 mM) was filter-sterilized, dispensed into aliquots, and stored at −20 °C.

^1^H NMR and ^31^P NMR spectra were recorded on a 500 MHz Bruker spectrometer equipped with a QXI probe and a ^31^P SEX probe, respectively. The compounds were suspended in D_2_O with sodium 3-trimethyl-1-propanesulfonate (TSP) which was used as an internal standard for ^1^H NMR, whereas 85% H_3_PO_4_ was used as an external standard for ^31^P NMR. Chemical shifts are given in parts per million (ppm), the letter “*J*” indicates spin-spin coupling constants which are given in Hertz (Hz).^1^H NMR (500.1 MHz,): δ: 6.97 (dt, 1H, ^1^*J*_HP_ 512.98 Hz, ^3^*J*_HH_ 1.75 Hz, H-P), 2.96 [dt ^3^*J*_HHa_ 7.47 Hz, ^3^*J*_HP_11.55 Hz, CH_2_-C(O)], 1.93 (m, 2H, CH_2_-P). ^31^P NMR (202.49 MHz, D_2_O, pH 7.02): δ: 28.13 (s). The NMR spectra of the studied compounds are shown in Fig. S5.

### Bacterial strain and culture media preparation

The wild-type *E. coli* K12 strain MG1655 (80) was used in this study and cultivated or resuspended in one of the following media, sterilized by autoclaving. The rich medium used was LB consisting of 10 g/l Bacto-Tryptone, 5 g/l Bacto-Yeast Extract, and 5 g/l NaCl dissolved in Milli-Q water and pH brought to 7.4 by dropwise addition of 5 M NaOH. The chemically defined medium was medium E, pH 7.0, which was prepared from a 50 times concentrated (50X) E medium (78), diluted to the working concentration (1X), having the following composition: 0.2 g/l MgSO_4_•7H_2_O, 2.0 g/l citric acid•H_2_O, 10.0 g/l anhydrous K_2_HPO_4_, 3.5 g/l NaNH_4_HPO_4_•H_2_O (11, 26) (https://www.uniroma1.it/en/brevetto/102016000098005). This was then supplemented with 20 g/100 ml (w/v) *D*-glucose to attain a final concentration of 0.4 g/100 ml and obtain the EG medium. The stock solution of *D*-glucose 20 g/100 ml (w/v) in Milli-Q water was sterilized separately by autoclaving for 15 min at 121 °C. The physiological solution was prepared by dissolving NaCl 0.9 g/100 ml (w/v) in Milli-Q water and sterilized by autoclaving. To revive cells, bacteria from 20% glycerol stocks stored frozen at −80 °C were streaked twice (*i.e.* on two consecutive days) on LB-agar plates and incubated at 37 °C overnight. For every assay, a pre-inoculum was carried out by transferring a single colony from freshly streaked bacteria on an LB-agar plate in LB medium and cultivating them by incubation at 37 °C, overnight and under vigorous shaking.

### Growth culture conditions and determination of minimum inhibitory concentration (MIC)

Bacterial growth curves and MICs were determined in chemically defined medium EG according to the microtiter broth dilution method as described elsewhere (26), using the microplate reader Biotek EL×808. The microplate was shaken for 30 s every 15 min to resuspend the bacteria. Briefly, overnight cultures (2 ml) of *E. coli* K12 strain MG1655 were centrifuged at 1500*g* for 15 min at 15 °C and the cell pellets resuspended in an isovolume of physiological solution (NaCl 0.9 g/100 ml, w/v) and the optical density at 600 nm (OD_600_) was brought to 1.0. The resuspended cells were diluted (1:25) in 2 ml of EG and grown at 37 °C under 150 rpm up to OD_600_ = 0.5 (7–8 h from the inoculum), then diluted again (1:25) in 3 ml of EG. This suspension was diluted 1:10 in the same medium in a 96-well microplate preloaded with serial dilutions of the compound(s) to be tested. The final volume in each well was 200 μl. The number of colony-forming units (CFU)/ml at time zero was between 0.5-1×10^6^, following the CLSI guidelines for antimicrobial susceptibility testing (81). For each compound tested, at least three independent assays were performed.

### Sample collection for metabolomic and proteomic analyses

Starting from overnight pre-cultures, the bacterial cultures to be subjected to omics analyses were prepared as described above, but with two key differences, *i.e.* the OD_600_ was brought to 2.0 and the first inoculum in EG medium was in 5 ml. When OD_600_ reached 0.5 (typically after 5 h), a 400 μl aliquot was used to inoculate 100 ml of EG medium (dilution 1:250), containing either *L*-Glu-γ-P_H_ or α-KG-γ-P_H_ at concentrations close to MIC_50_, *i.e.* 3.5 μg/ml and 8.5 μg/ml, respectively. For the untreated samples, a 280 μl aliquot was diluted 1:10 in 2.52 ml of EG medium and then 500 μl was used for the inoculum in 100 ml of EG medium (total dilution 1:2000). The eight times higher dilution in the control culture was necessary to ensure that after overnight growth it reached the same OD_600_ 1.0 ± 0.1, as the treated cultures. Two biological replicates, from two independent pre-cultures, were used each time to inoculate a total of six flasks, *i.e.* two flasks for each experimental condition (*i.e.* control/untreated cells, *L-*Glu-γ-P_H_ - and α-KG-γ-P_H_ -treated cultures). This was carried out 5 times to obtain a total of ten different biological replicates for each experimental condition. A sixth additional experiment was performed with four biological replicates of the control condition because in two of the above experiments, after the overnight growth, the untreated/control reached OD_600_ 1.78 to 2.2, and therefore the OD_600_ was considered not suitable in the downstream analysis.

The experimental workflow is depicted in Fig. S2. Bacteria cultures were incubated at 37 °C, 150 rpm until the late-exponential phase (OD_600_ = 1.0 ± 0.1), which was reached after 17.5 ± 0.9 h, 25.2 ± 2.8 h and 23.5 ± 2.5 h for the untreated cultures, the *L-*Glu-γ-P_H_ -treated and the α-KG-γ-P_H_ -treated cultures, respectively. For the metabolomic and proteomic analyses, samples were split in two 50-ml tubes and immediately transferred to a −35 °C bath (Julabo FP50-HE) for 2.5 min, for metabolism quenching. Then, the tubes were centrifuged for 5 min at 5000*g* at 4 °C, to obtain the bacterial pellets to which two different protocols were applied to prepare samples for metabolomic and proteomic analysis.

#### Metabolomic studies

The cell pellets were washed in 1.5 ml of ice-cold physiological solution, then transferred in a 2-ml microcentrifuge tube and centrifuged for 5 min at 5000*g* at 4 °C; the supernatants were discarded, and each pellet was resuspended in 1 ml of ice-cold physiological solution. The OD_600_ was measured using a 5-μl aliquot diluted 1:200. Based on the OD_600_ readings, the volume corresponding to OD_600_ = 25 (∼350 μl) was transferred to another 2 ml microcentrifuge tube and centrifuged for 5 min at 5000*g*, at 4 °C. The physiological solution was discarded, and the pellets were immediately frozen in liquid nitrogen and stored at −20 °C.

#### Proteomic studies

The cell pellets were resuspended in 1 ml of ice-cold physiological solution and transferred to 2 ml microcentrifuge tubes. Samples were resuspended in ice-cold physiological solution and centrifuged twice for 5 min at 5000*g*, at 4 °C to achieve complete removal of the residual spent medium; then the weight of each pelleted material was measured (102.7 ± 8.5 mg) and the samples were immediately frozen and stored at −20 °C.

### Metabolite extraction, NMR data collection, and processing

Pellets stored for metabolomics studies were thawed on ice, resuspended in 750 μl of ice-cold methanol (60% v/v), submitted to three freeze-thaw cycles using liquid nitrogen, and centrifuged for 5 min at 12,900*g*, at 4 °C. The supernatants were transferred into 1.5-ml microcentrifuge tubes, and the extraction procedure was repeated a second time on the residual pellet. Samples were then concentrated down to dryness using a speed-vacuum concentrator (Vacufuge plus, Eppendorf) and stored at −20 °C until use. Dried extracts were dissolved in 250 μl of 0.35 M potassium phosphate buffer, pH 7.0, 451.5 μl D_2_O with 2 mM sodium azide, and 50 μl D_2_O with 3.2 mM TSP (final volume 751.5 μl per sample). Subsequently, 600 μl of each sample was transferred to a 5 mm NMR tube for ^1^H NMR spectra acquisition. For ^31^P NMR, after ^1^H NMR acquisition, samples were pooled, concentrated by speed-vacuum concentrator (as above) dissolved in 1 ml of D_2_O, and transferred to a new 5 mm tube. Prior to ^31^P NMR spectra acquisition, samples were checked for pH, which ranged between 7.1 to 7.4.

^1^H NMR spectra were acquired on a Bruker Avance II^+^ 800 MHz spectrometer equipped with a 5 mm TCI H&F/C/N/D cryoprobe. All 1D ^1^H were acquired at 298.15 K and using a *noesygppr1d* pulse program, in which water pre-saturation occurred during mixing time and relaxation delay. Acquisition parameters were the following: 80 scans, relaxation delay of 4 s, mixing time of 10 ms, spectral width of 20.0237 ppm. The size of free induction decay (FID) was 128k points. Processing of spectra was performed with Bruker TopSpin 3.2. All FIDs signals were multiplied by an exponential function, followed by Fourier Transformation. Spectra were manually phase and baseline corrected. For the spectral assignment, 2D NMR spectra were acquired for some samples: ^1^H-^1^H TOCSY (*dipsi2esgpph* pulse sequence, 512 points in F1 and 2048 points in F2; 128 scans; relaxation delay of 1.5 s; mixing time of 60 ms; sweep width of 8012.12 Hz for both dimensions); ^1^H-^13^ C HSQC (*hsqcetgpsisp2* pulse sequence, 512 points in F1 and 2048 points in F2; 128 scans; relaxation delay of 1.5 s; sweep width of 33,207.441 Hz in F1 and 12,820.513 Hz in F2) and ^1^H J-resolved (*jresgpprqf* pulse sequence, 100 points in F1 and 8192 points in F2; 16 scans; relaxation delay of 2 s; sweep width of 78.113 Hz in F1 and 133,68.984 in F2).

^31^P NMR spectra were acquired on a Bruker Avance II 500 MHz spectrometer equipped a 5 mm SEX P-H-D probe. ^31^P NMR spectra were acquired at 298.15 using a proton decoupled *zgpg* pulse program. Acquisition parameters are the following: 1000 scans, relaxation delay of 1 s, spectral width of 99.5630 ppm. The size of free induction decay (FID) was 128k points.

Processing of spectra was performed with Bruker TopSpin 3.2. All processed 1D ^1^H NMR spectra were grouped and transformed into a matrix, *i.e.* each column was a spectrum, while each row was one of the 128k points that make up the FID. The spectra were aligned and calibrated according to the chemical shift of TSP at 0.00 ppm. For the untargeted analysis, the region of water (4.70–4.95 ppm) was removed. After trimming the interfering peaks, only the region between 0.15 to 10.00 ppm was used. All aforementioned steps were done according to standard procedure and completed using an R software environment for statistical computing (v3.6.2) with homemade scripts (82). The generated matrix was used to assess the presence of outliers in the experimental groups.

The Chenomx NMR suite 8.12 (Chenomx, Edmonton, Alberta, Canada) was used for the identification and quantification of individual metabolites using the Chenomx reference library for 800 MHz NMR and the Human Metabolome Database (HMDB) library.

### Proteomic analysis and mass spectrometry proteomic data

Each bacterial pellet was resuspended in LDS 1X (Invitrogen) consisting of 106 mM Tris-HCl, 141 mM Tris-base, 10% glycerol, 2% lithium dodecyl sulfate, 0.51 mM EDTA, 0.175 mM phenol red, and 0.22 mM SERVA Blue G250, buffered at pH 8.5 and supplemented with 2.5% ß-mercaptoethanol. The volume of LDS 1X used was 12.5 μl per mg of pellet. Cells were lysed by incubation for 10 min at 99 °C followed by 30 s sonication with a disruptor set at 80% amplitude with impulsion of 1 s every 4 s, and bead-beating lysis with a mixture of beads (0.1 mm silica beads, 0.1 and 0.5 mm glass beads) in a Precellys Evolution instrument (Bertin technologies) as recommended (83). After centrifugation at 16,000*g* for 3 min, the supernatants were collected, diluted with four volumes of LDS 1X, and then heated at 99 °C for 5 min. For each sample, the proteins contained in a volume of 15 μl (corresponding to 0.24 mg of the bacterial pellet) were subjected to electrophoresis on a NuPAGE 4 to 12% Bis-Tris (Invitrogen) gel. After 5 min migration at 200 V in MES buffer (Invitrogen), the proteins were stained with ready-to-use Coomassie SimplyBlue SafeStain (Thermo Fisher Scientific), excised as a single polyacrylamide band, treated and proteolyzed with trypsin as previously described (84). A fraction of the resulting peptide pool (5 μl out of 50 μl) was analyzed by tandem mass spectrometry with a Q-Exactive HF instrument (Thermo Fisher Scientific) in similar conditions as those previously described (85). Peptides were desalted online with a PepMap 100 C18 pre-column and then resolved on a reverse-phase Acclaim PepMap 100 C18 column (500 mm length, 3 μm granulometry, 100 Å porosity, 75 μm inner diameter, 450 m^2^/g specific surface; Thermo Fisher Scientific) at a flow rate of 200 nl/min with a 90 min gradient (4–25% B from in 75 min, and then 25–40% B from 15 min) with mobile phases A (0.1% HCOOH/100% H_2_O) and B (0.1% HCOOH/80%CH_3_CN/19.9%H_2_O) delivered by an Ultimate 3000 chromatography system (Thermo Fisher Scientific). The mass spectrometer was operated in data-dependent acquisition mode with a Top20 strategy consisting of cycles of a full scan of peptide ions followed by sequential fragmentation of each of the 20 most intense precursors in the high energy collisional dissociation cell and MS/MS scans of the resulting fragments. Only peptide ions with a charge state of 2+ or 3+ were selected for dissociation, with a dynamic exclusion of 10 s. Full scan mass spectra from 350 to 1500 *m/z* were acquired at a resolution of 60,000 while MS/MS scans were recorded at a resolution of 15,000. Peptide-to-spectrum assignation was performed with the Mascot software v2.5.1 (Matrix Science) against the *E. coli* K12 database (downloaded 2020/04/30, 4545 entries totaling 1,428,385 residues) with the following parameters: full-trypsin specificity with up to two missed cleavages allowed, fixed modification of carbamidomethylated cysteine, mass tolerances of 5 ppm for peptide search and 0.02 Da for peptide fragments. Variable modifications were the oxidation of methionine and deamidation of asparagine and glutamine. Peptide matches with a Mascot peptide score below a *p*-value of 0.05 were selected. Proteins were considered valid when at least two different peptides were detected. False Discovery Rate (FDR) for protein identification was below 1% as estimated with the reverse database decoy Mascot search option. Spectral counts were compared between conditions after standard normalization using the T-Fold method as described (33), selecting proteins that satisfied |T-fold| (≥1.5) and *p*-value (≤0.05).

Mass spectrometry proteomics data have been deposited to the ProteomeXchange Consortium *via* the PRIDE partner repository under the dataset identifiers PXD051418 and 10.6019/PXD051418.

### Multivariate, univariate, and bioinformatics data analyses

The multivariate statistical analysis of metabolomics and proteomics data was performed using the Metaboanalyst Analytical Pipeline (34, 86).

For metabolomics, missing values were replaced with half of the minimum positive value found within the original data as explained elsewhere (34). Metabolite concentrations were autoscaled whereas protein spectral counts were pareto scaled, without normalization or transformation (34, 86). Multivariate analysis was performed, namely Principal Component Analysis (PCA) and Partial Least Squares-Discriminant Analysis (PLS-DA). The PLS-DA model was validated through cross-validation (Q^2^) and permutation tests (separation distances, B/W), and the model was considered valid when the permutation test gave a *p*-value < 0.05. To understand which metabolites were contributing the most to the separation observed in the PLS-DA, variables were then ranked based on the Variable Importance in Projection (VIP) scores. Heatmaps were built using Euclidean distance measure and Ward clustering algorithm.

Metabolites responsible for the discrimination between groups of samples were also analyzed by univariate analysis, concentration values 4 times above/below the InterQuartile Range (IQR) were considered outliers and excluded from the analysis. The assumption of normality was assessed by Shapiro-Wilk’s test and, based on the results, the one-way analysis of variance (ANOVA) and Kruskal-Wallis’s test were used as parametric and nonparametric tests, respectively. Statistical significance was defined as *p*-value < 0.05. To correct for pairwise multiple comparisons and adjust the *p*-values, the *post hoc* Tukey’s HSD test or Mann-Whitney U’s test (with the Bonferroni’s correction) were used.

For proteomics data univariate analysis, spectral counts were compared between conditions after standard normalization using the T-Fold method as described (33), selecting proteins that satisfied |T-fold| (≥1.5) and *p*-value (≤0.05). These proteins were also subjected to a Gene ontology (GO) enrichment analysis using the Gene Ontology Consortium’s online tool (http://www.geneontology.org/) (87). First, from the UniProKB codes the gene names of each protein were retrieved, and two gene lists were created: one with gene names of proteins whose T-fold (TF) was ≥ 1.5, and one with gene names of proteins whose TF was ≤ −1.5 compared to the control group. Then, each gene list was submitted to the GO tool (87), and the GO enrichment analysis for the biological process was performed selecting *E. coli* as a species. For statistical analysis, Fisher’s exact test was used, and *p*-values were corrected using the Benjamini-Hochberg FDR’s test. Only GO-enriched terms with a corrected *p*-value < 0.05 were considered significant.

The integration of the metabolomics and proteomics data was performed using the Joint-Pathway Analysis module of the Metaboanalyst Analytical Pipeline (34). Only metabolites with statistically significant different amounts (*p*-value < 0.05) relative to the control treatment and proteins with statistically significant amounts (*p*-value < 0.05) with a TF > 1.0 and < −1.0 relative to the control treatment were used. For each metabolite, the KEGG ID was retrieved, while for each protein the UNIPROT ID was used, along with the relative TFs. Then, the metabolite and protein lists were submitted selecting the *E. coli* K-12 strain MG1655 as a species, and the analysis was carried out using the Metabolic pathways as Pathway database. For the Enrichment analysis, the Hypergeometric test was used, while for the Topology measure and the Integration method, the default options (*i.e.* degree centrality and combine queries, respectively) were selected.

## Data availability

Mass spectrometry proteomics data have been deposited to the ProteomeXchange Consortium *via* the PRIDE partner repository under the dataset identifiers PXD051418 and 10.6019/PXD051418. NMR metabolomic data is available at the NIH Common Fund’s National Metabolomics Data Repository (NMDR) website, the Metabolomics Workbench, https://www.metabolomicsworkbench.org where it has been assigned Study ID ST003202 ('Metabolomics Workbench: An international repository for metabolomics data and metadata, metabolite standards, protocols, tutorials and training, and analysis tools (2016)' [PubMed: https://www.ncbi.nlm.nih.gov/pubmed/26467476/].). The data can be accessed directly *via* its Project DOI: https://doi.org/10.21228/M8QH90.

## Supporting information

This article contains supporting information.

## Conflict of interests

The authors declare that they have no conflicts of interest with the contents of this article.

## Supporting information

Supplementary Table S4

Suppelementary Table S5
