# Supplementary material for: Integrated multi-omics unveil the impact of H-phosphinic analogs of glutamate and α-ketoglutarate on Escherichia coli metabolism
Source: J Biol Chem. 2024 Sep 21;300(10):107803. doi: 10.1016/j.jbc.2024.107803 (PMC11533085; doi:10.1016/j.jbc.2024.107803)
Supplement: Supplementary Figure [file mmc1.pdf]

Supplementary Figures

Giovannercole et al.

«Integrated multi-omics unveil the impact of *H*-phosphinic analogues of glutamate and α-ketoglutarate on *Escherichia coli* metabolism»

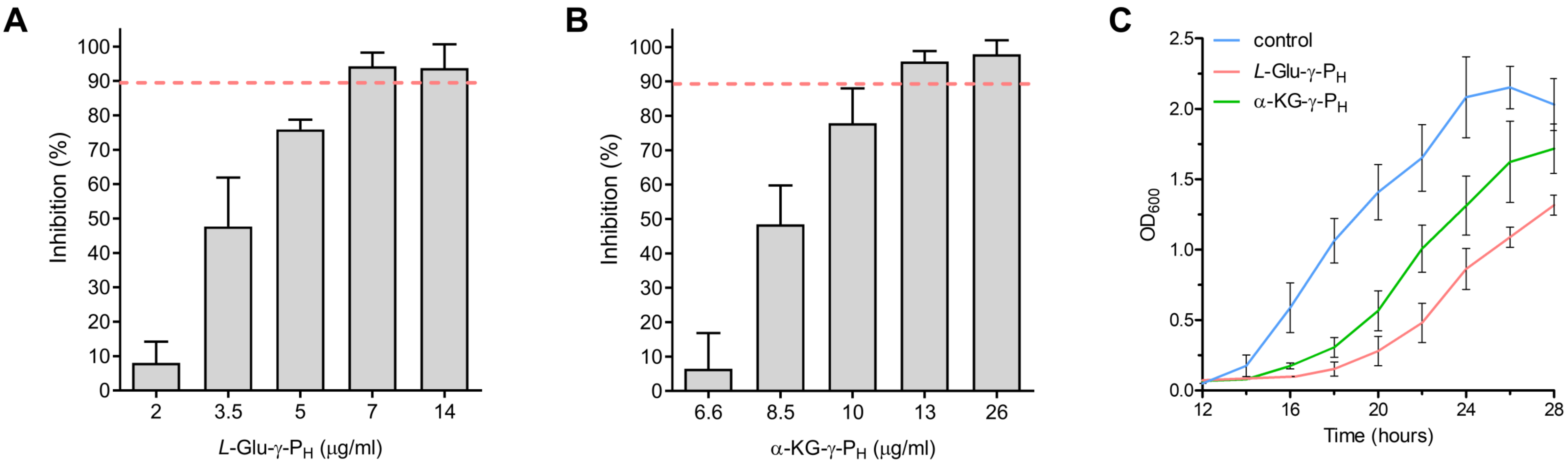

Figure S1. *Assessment of the inhibitory activity of* L-*Glu-γ-*P_H *and α-KG-γ-*P_H. **(A, B)** *E. coli* cells were exposed to different concentrations of *L*-Glu-γ-P_H **(A)** and α-KG-γ-P_H **(B)** in EG medium pH 7.0 and incubated at 37 °C. Cells not exposed to neither of the two compounds (untreated) were grown in parallel as a negative control. After 22 hours of growth, the OD_{600} was determined, and the inhibitory activity was derived by the following equation:inhibition (%) = [1 − (OD_{600}treated/OD_{600}untreated)] × 100. The MIC_{90} was calculated as the lowest tested concentration of *L*-Glu-γ-P_H and α-KG-γ-P_H that inhibits 90% (dashed line in red) of the growth. **(C)** *E. coli* growth curves in EG pH 7.0 with sub-inhibitory (MIC_{50}) concentrations of *L*-Glu-γ-P_H (3.5 μg/ml), α-KG-γ-P_H (8.5 μg/ml) or neither of the two compounds (control). In A, B and C, data are displayed as mean ± standard deviation from at least 3 independent experiments.

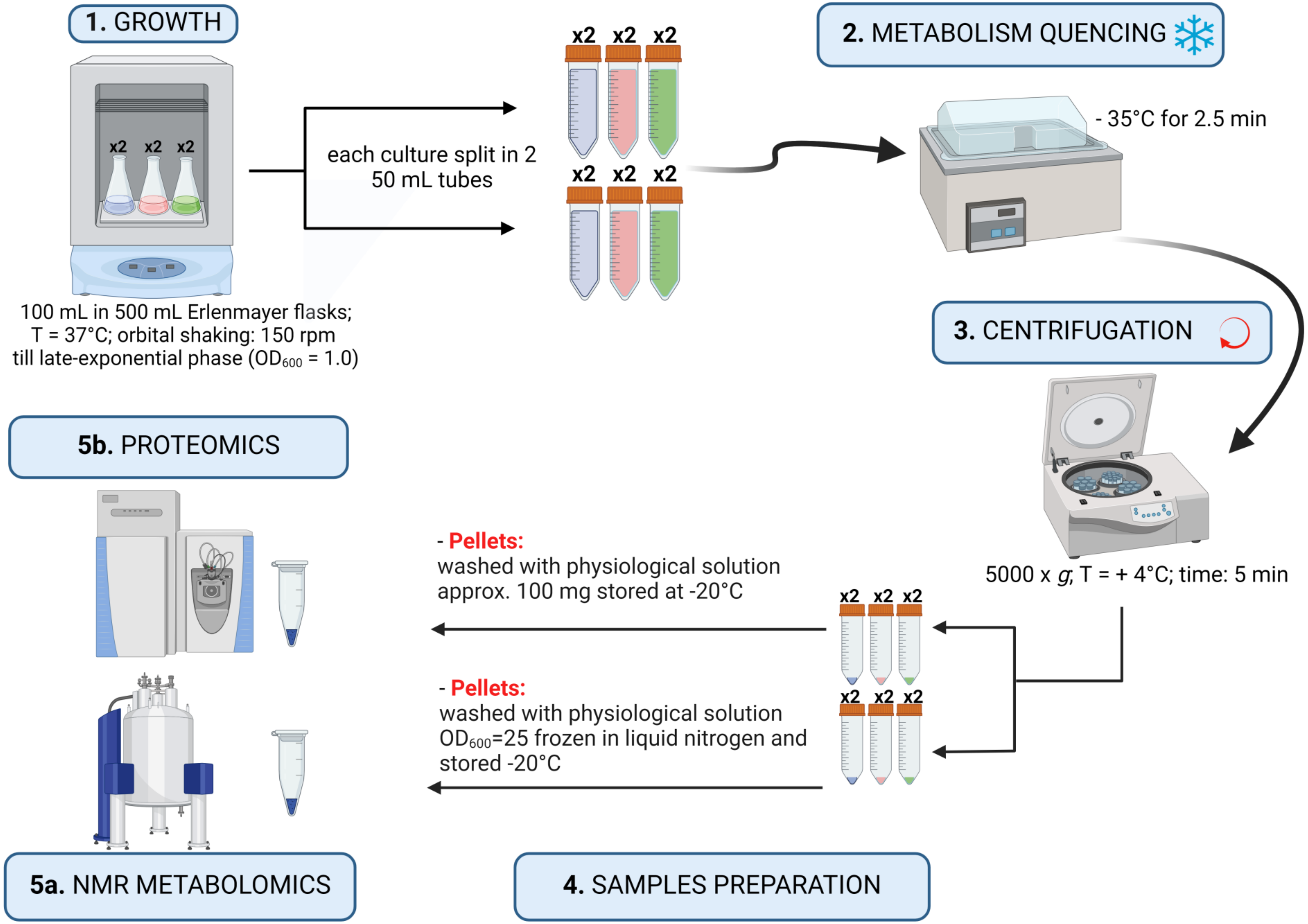

Figure S2. *Experimental workflow for sample processing for metabolomics and proteomics analyses* (created with BioRender.com). The biological replicates of each condition, i.e. untreated control group (light blue), *L*-Glu-γ-P_H-treated group (pink) and α-KG-γ-P_H-treated groups (green) were collected and processed according to this depicted workflow.

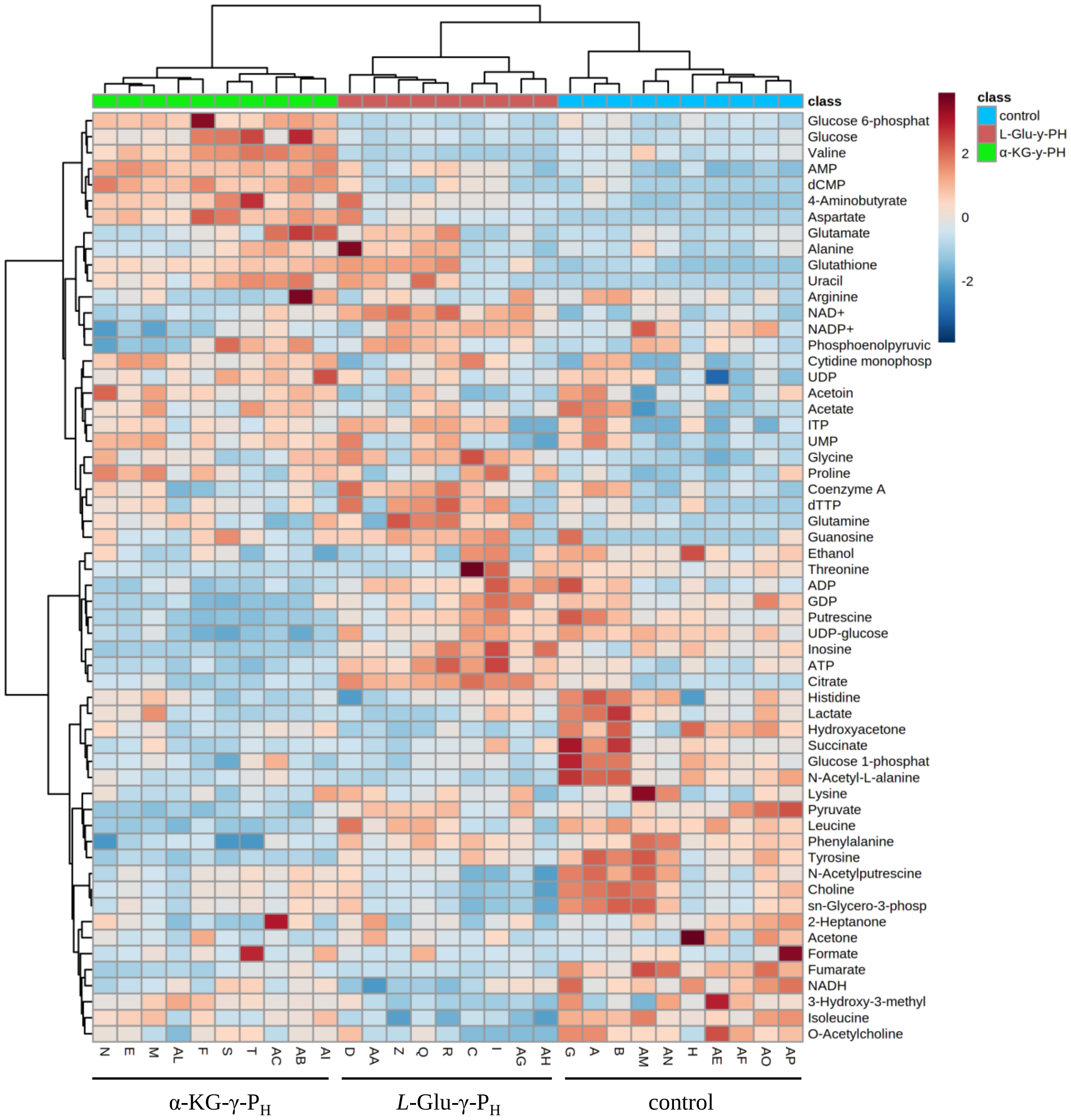

Figure S3. *Heatmap visualization for all the metabolites observed by ^{1}H NMR.* Biological replicates (horizontal axis) and single metabolites (vertical axis) are separated using a hierarchical clustering based on Euclidean distance.

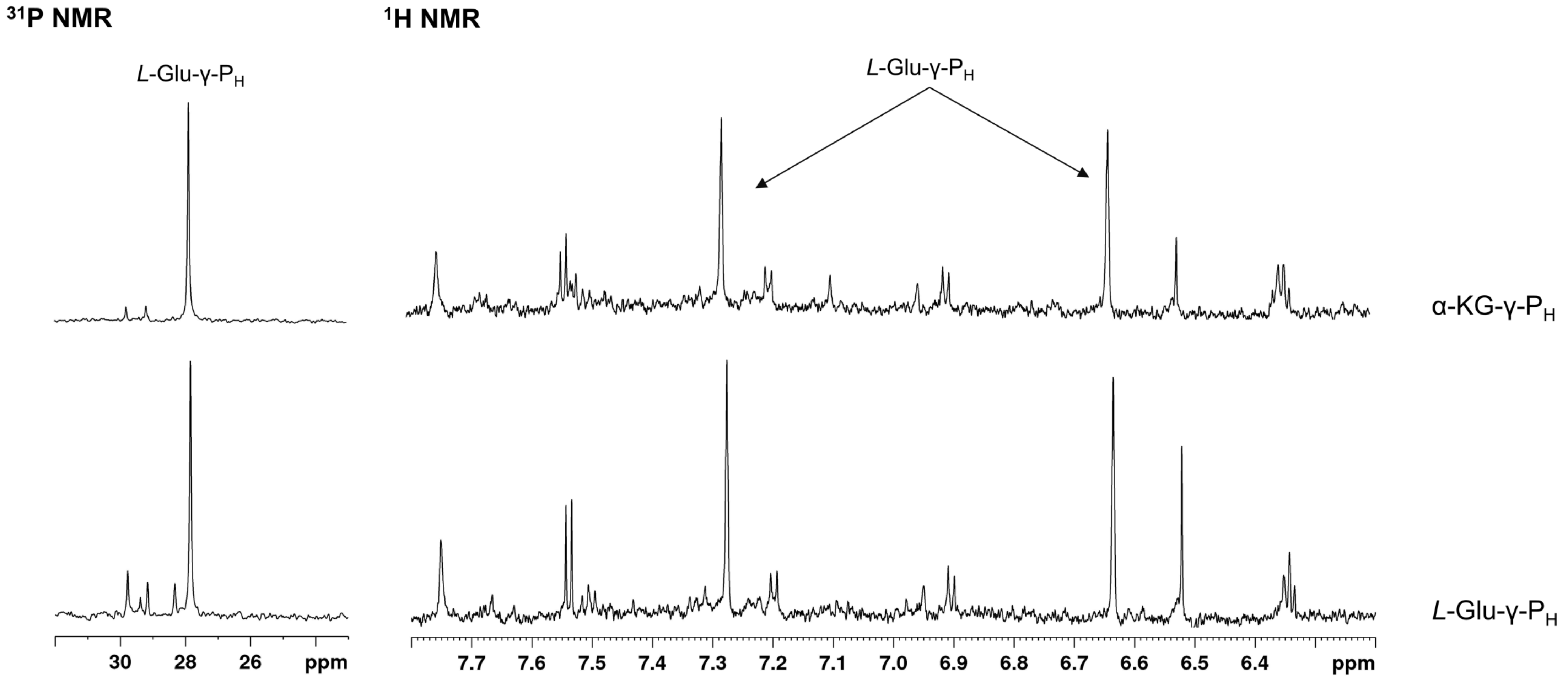

Figure S4. *Highlight of the ^{31}P NMR and ^{1}H NMR spectra of* E. coli *incubated with the phosphinic compounds.* In the case of the ^{31}P NMR spectra, it is a combination of the different extracts incubated with *L*-Glu-γ-P$_H$ (spectra below) and with α-KG-γ-P$_H$ (spectra above). The *L*-Glu-γ-P$_H$ resonances are indicated in the figure.

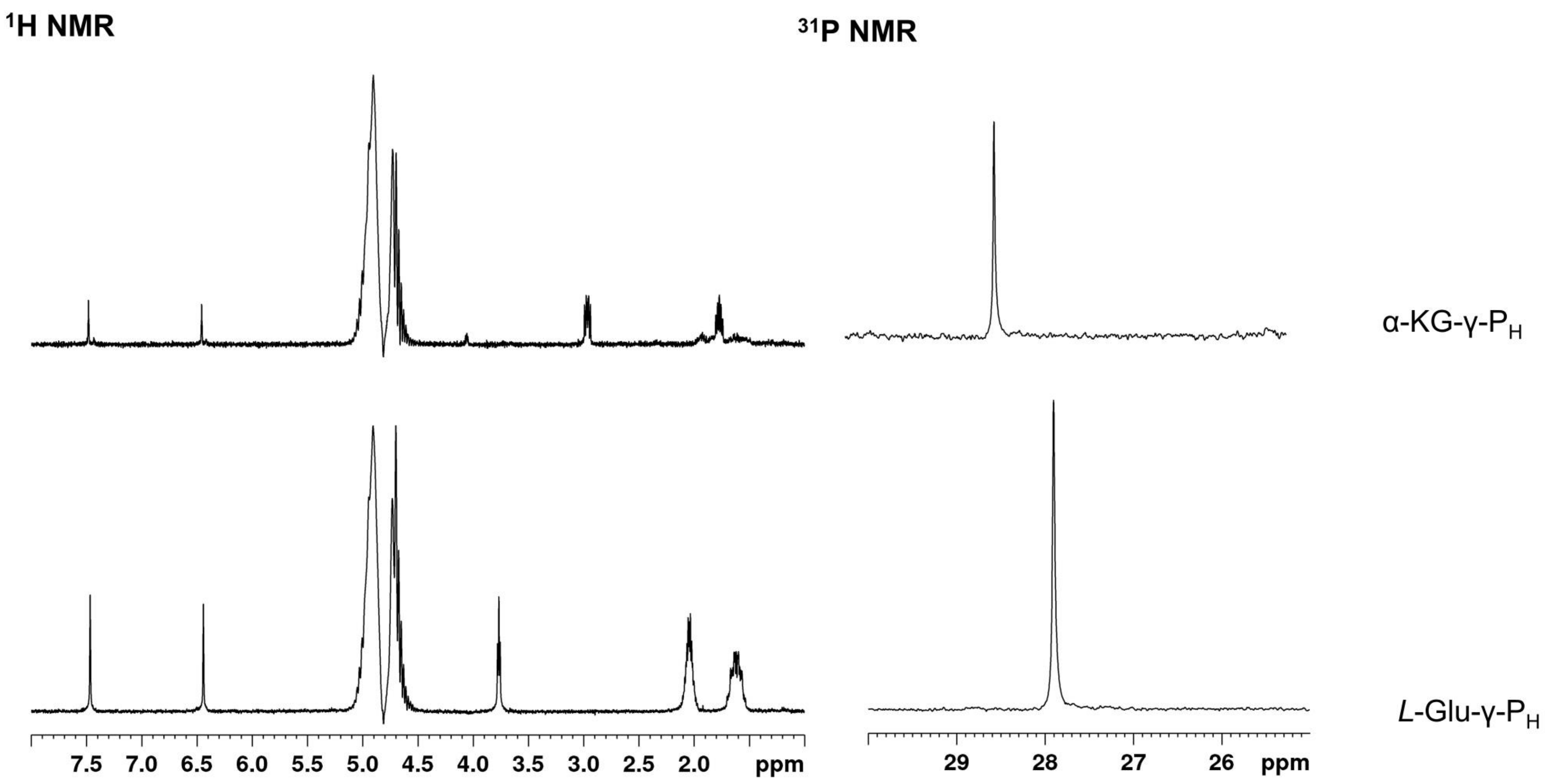

Figure S5. *Highlight of the ^{31}P and ^{1}H NMR of the phosphinic compounds.* ^{1}H (left) and ^{31}P (right) NMR spectra of the α-KG-γ-P_H (top) and *L*-Glu-γ-P_H (bottom).
